# Precision targeted ruthenium(ii) luminophores; highly effective probes for cell imaging by stimulated emission depletion (STED) microscopy†
†Electronic supplementary information (ESI) available: Detailed synthesis and characterisation of metal complexes and peptides. See DOI: 10.1039/c6sc02588a



**DOI:** 10.1039/c6sc02588a

**Published:** 2016-06-30

**Authors:** Aisling Byrne, Christopher S. Burke, Tia E. Keyes

**Affiliations:** a School of Chemical Sciences , National Centre for Sensor Research , Dublin City University , Dublin 9 , Ireland . Email: tia.keyes@dcu.ie; b National Biophotonics and Imaging Platform , DCU , Ireland

## Abstract

Fluorescence microscopy has undergone a dramatic evolution over the past two decades with development of super-resolution far-field microscopy methods that break the light diffraction limited resolution of conventional microscopy, offering unprecedented opportunity to interrogate cellular processes at the nanoscale. However, these methods make special demands of the luminescent agents used for contrast and development of probes suited to super-resolution fluorescent methods is still relatively in its infancy. In spite of their many photophysical advantages, metal complex luminophores have not yet been considered as probes in this regard, where to date, only organic fluorophores have been applied. Here, we report the first examples of metal complex luminophores applied as probes for use in stimulated emission depletion (STED) microscopy. Exemplified with endoplasmic reticulum and nuclear targeting complexes we demonstrate that luminescent Ru(ii) polypyridyl complexes can, through signal peptide targeting, be precisely and selectively delivered to key cell organelles without the need for membrane permeabilization, to give high quality STED images of these organelles. Detailed features of the tubular ER structure are revealed and in the case of the nuclear targeting probe we exploit the molecular light switch properties of a dipyrido[3,2-*a*:2′,3′-*c*]phenazine containing complex which emits only on DNA/RNA binding to give outstanding STED contrast and resolution of the chromosomes within the nucleus. Comparing performance with a member of the AlexaFluor family commonly recommended for STED, we find that the performance of the ruthenium complexes is superior across both CW and gated STED microscopy methods in terms of image resolution and photostability. The large Stokes shifts of the Ru probes permit excellent matching of the stimulating depletion laser with their emission whilst avoiding anti-Stokes excitation. Their long lifetimes make them particularly amenable to gated STED, giving a much wider window for gating than traditional probes. Our findings indicate that ruthenium polypyridyl peptide targeted probes are a powerful new partner to STED microscopy, opening up new approaches to probe design for STED microscopy.

## Abstract 2

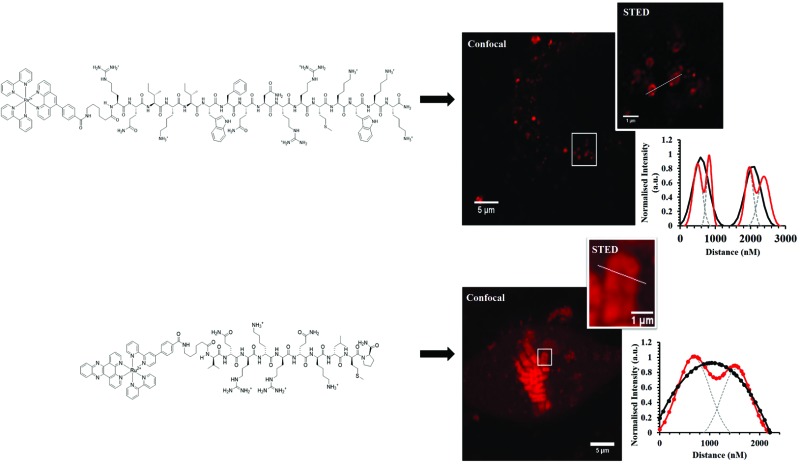
Using precision peptide targeting to discrete cell organelles, it is demonstrated that Ru(ii) polypyridyl complexes are highly effective probes for stimulated emission depletion microscopy.

## Introduction

Fluorescence microscopy is the key bioimaging tool used to study live cells, tissues and other biological structures. Whereas the spatial resolution of classical far field microscopy is restricted by the light diffraction limit, over the past two decades a number of super-resolution optical microscopy methods have been advanced which break the diffraction limit.^1–8^ The first and probably the best known of these methods is STimulated Emission Depletion (STED) microscopy which works by depleting a zone around the edge of the focal region of emitting fluorophore.^9^ This is achieved by switching the fluorophore in the depletion zone to a dark state by stimulating its emission with a high intensity laser that matches the emission wavelength (usually the red edge of the emission) of the fluorophore. This effectively reduces the spot size of the incident laser to below the diffraction limit. For STED, sub-50 nm resolution has been reported.^10^ Each of the super resolution optical methods to advance over recent years has different advantages and disadvantages.^11^ Key strengths of STED are; the resolution improvement is molecular, thus, data does not typically need additional post-processing, STED has achieved the lowest resolution at sub-50 nm of all of the super-resolution methods.^2^ Optical sectioning is intrinsic to the STED method enabling the acquisition of planes of roughly 100 nm and 3-dimensional structures even several tens of microns deep inside the tissue have been achieved.^3^ Relatively fast image acquisition of several images per second means that it can be used on live samples. Consequently, STED has been used to non-invasively image organelle structures and molecular dynamics at the nano-scale,^12^ for example to image nerve and synaptic tissues in neuronal cultures^13^ and dendritic spine synaptic signals.^14^

**Table 1:** Summary of photophysical and STED full width half maxima (FWHM) data for RuER and RuArg_8_ carried out in PBS solution

Complex	*Φ* (%)	*τ*_aerated_ 18 °C (ns)	*τ*_de-aerated_ 18 °C (ns)	FWHM_confocal (nm)_	FWHM_STED (nm)_
RuArg_8_	0.067 ± 0.005^*a*^	579 ± 11	1.02 μs ± 9 ns	226.66	86.8
RuER	0.0316 ± 0.0015^*a*^	683 ± 8	1.06 μs ± 14 ns	532.44	151.72

^*a*^Standard used [Ru(bpy)_3_]^2+^ 0.04 ± 0.002.

Because a molecular mechanism underlies the operation of STED, the photophysics of the luminophore bears an important influence on performance. The lateral resolution, Δ*x*, achieved in STED depends on the efficiency of the dye de-excitation by stimulated emission according to eqn (1).1
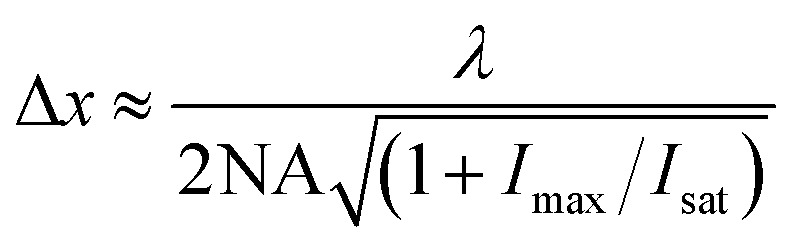
where NA is the numerical aperture of the objective, *I*_max_ is the peak intensity of the STED laser (*i.e.* peak intensity within the depletion zone) and *I*_sat_ is the applied STED laser threshold intensity, *i.e.* the intensity that yields 50% depletion of the probe emission intensity and *λ* is the emission wavelength. As eqn (1) indicates, the more efficient the depletion process the greater the achievable resolution.^15^ And it follows that the more intense the STED laser the greater the depletion. Thus, STED makes substantial demands on the fluorescent probe beyond conventional fluorescence microscopy. Super-resolution achieved by depleting a fluorescent probe (fluorescent lifetime 1 to 10 ns) typically demands 0.1–1 GW cm^–2^ from a CW depletion source therefore the fluorophore must be photo-stable when irradiated at the depletion wavelength. Furthermore, the STED depletion laser wavelength must be capable of stimulating emission whilst avoiding promoting the probe to a long-lived dark state or inducing bleaching or photo decomposition.^16^ The more closely matched the depletion laser to the emission maxima of the probe molecule, the more efficient the stimulated emission. In practice, because of the small Stokes shifts of typical organic fluorophores the STED laser wavelength is matched to the red tail of the emission spectrum to avoid overlap with the dye absorption spectrum and thus re-excitation or excitation into a photoactive state which leads to a prolonged dark state such as a triplet–triplet absorbance or to photobleaching. This is not ideal since, as described, depletion improves the greater the overlap of the STED laser with the emission spectrum, as the higher the stimulated absorption cross section will be. Furthermore, in STED one of the most important issues for optimization of the stimulated emission is the time-delay between excitation and depletion lasers. The STED laser beam should impinge the sample whilst the probe resides in its excited state and the pulse width of the stimulated de-excitation laser must be narrower than the excited state lifetime of the probe molecule.

In summary, a good STED probe should exhibit high intensity red to NIR emission, good photostability, a long lived emissive state and good overlap of emission spectrum with the STED laser whilst avoiding re-excitation through ground state absorbance or promotion of the compound through excited state absorption to a long-lived dark state/photobleach. It should also be capable of permeating the cell membrane and in particular it should target with high precision and selectivity, the site of interest.

Most luminescent probes used in microscopy, and all used in super-resolution microscopy to date, are organic, they typically have short emission lifetimes, small Stokes shifts and are frequently prone to photodecomposition. A number of commercial fluorescent dyes are recommended for STED imaging namely AlexaFluor Fluor, Atto and Chromeo dyes^17–21^ as these are very photostable compared with traditional fluorescent probes such as fluorescein.^22^ There is now growing interest in expanding the repertoire of probes available to STED microscopy and a number of organic fluorophores have been devised to address individually some of the key issues highlighted above, most notably red or NIR emission, Stokes shift, photostability and permeability.^23–27^ However, no probe has been reported which combines all of the elements of a good STED probe.

Transition metal complex luminophores such as those based on Ru(ii) polypyridyl complexes, the focus of this study, are red-emitting probes which exhibit a number of important properties which we anticipated might make them a useful alternative to organic probes for exploitation in STED microscopy. The most important are their: large Stokes shift, long lived emissive states and good photostability.^28^ Formally, emission from a Ru(ii) polypyridyl complex is a phosphorescence originating from a triplet metal to ligand charge transfer state that forms rapidly (<300 fs) and with unit efficiency following singlet excitation enabled by strong-spin orbit coupling from the metal centre.^29^ Such complexes emit at room temperature with relatively high quantum yields, but with peak to peak separation between their absorbance and emission maxima of between 120 and 200 nm. This means that effective overlap of the depletion laser with the emission spectrum is enabled without the possibility of re-absorption by the ground state of the dye. In addition, stimulated emission from the phosphorescent state can be observed in transient spectroscopy of the ruthenium complexes whereas only weak transient absorption is typically observed in the region of stimulated emission.^16^ Furthermore, large Stokes shifts allow their study in microscopy at high concentrations without dye performance being compromised by self-quenching, so such complexes can be accumulated at high concentration into small volumes such as organelles.

Organic fluorophores typically have emission decays in the range of 500 ps to 5 ns, which set a very narrow limit on the pulse width of the depletion laser, typically to 200 or 300 ps. Whereas, by extending the probes lifetime one increases the probability of stimulated emission and so of achieving improved resolution by STED. Ruthenium polypyridyl complexes exhibit long lived emitting states, typically between 200 and 1000 ns, so not so long lived that they overly slow the cycle time for STED. As described, STED, because of the use of a high intensity source used to switch the probe to a dark state makes particular demands on the photostability of the probe and, ruthenium polypyridyl and related complexes typically show very good photostability compared with organic fluorophores.^30^ In spite of all of these potential benefits there have, to date, been no reports of Ru(ii) polypyridyl or indeed any related metal complex luminophores applied to super-resolution imaging.

**Fig. 1:**
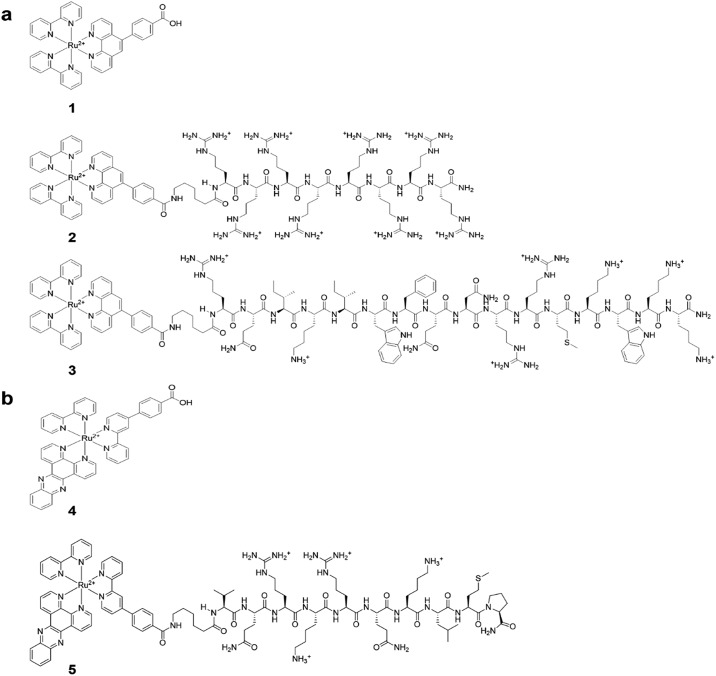
(a) Structures of [(Ru(bpy)_2_-phen-Ar)-COOH]^2+^ (**1**), Ru-Arg_8_ (**2**), Ru-ER (**3**). (b) Structures of [Ru(dppz)(bpy)(bpyArCOOH)]^2+^ (**4**), and Ru-NLS (**5**).

Conversely, there is strong emerging interest in the application of metal complex luminophores to confocal fluorescence/luminescence imaging because of their aforementioned photophysical properties and established on extensive studies on their DNA interactions.^31–43^ Our group and others have been studying the application of Ru(ii) peptide conjugated probes to imaging and sensing in live cells and have demonstrated that cell penetrating peptide (CPP) sequences are effective in driving metal complexes, which are ordinarily membrane impermeable, across the plasma membrane.^30,44^ Initial studies also indicate that peptide signals are very effective at driving Ru(ii) polypyridyl complexes to discrete organelles such as the mitochondria.^45–48^ Herein, we report on the application of ruthenium polypyridyl complexes for the first time as STED probes. Using two novel organelle targeted ruthenium polypyridyl complex peptide conjugates [Ru(bpy)_2_-phen-Ar-ER]^9+^, known as Ru-ER from here on in, and [Ru(dppz)(bpy)-bpy-Ar-NLS]^6+^ (Ru-NLS), as well as non-targeted cell penetrating peptide (CPP) octaarginine conjugate, [Ru(bpy)_2_-phen-Ar-Arg_8_]^10+^(Ru-Arg_8_), shown in Fig. 1, we examine the capacity of these complexes to both direct the complexes to discrete organelles and therein their effectiveness as STED probes. We demonstrate that such complexes combine both appropriate photophysics and the capacity to be highly selectively targeted to key organelles within the living cell to enable STED imaging of such targets. Comparing their behaviour to a commonly recommended commercial Probe for STED, AlexaFluor Fluor 532,^20,49,50^ we show that these complexes are useful alternative STED probes yielding remarkably high contrast images with approximately 2-fold improvement in resolution compared with AlexaFluor Fluor 532 under the experimental conditions used here and with photostability comparable to AlexaFluor Fluor 532.

## Results and discussion

### Syntheses and characterization of ligands and complexes

[Ru(bpy)_2_-phen-Ar-ER]^9+^ **3** and [Ru(bpy)_2_-phen-Ar-Arg_8_]^10+^ **2** and associated parent complex, **1**, were prepared according to modifications of previously reported protocols.^48^ [Ru(dppz)(bpy)-(bpy-Ar-NLS)]^6+^, **5**, is a tris–heteroleptic complex which was prepared *via* a high yield (>80%) 5 step synthesis recently reported which lead to unconjugated parent complex which was then conjugated to the NLS peptide through a HBTU coupling protocol as described in ESI†.^51^

The nuclear localizing sequence (NLS) and endoplasmic reticulum directing (ER) sequences exploited here are derived from natural signal peptide sequences. In the cell, signal sequences are used to facilitate migration of protein, following their synthesis in the cytoplasm, to their target organelles^52^ and signal peptides have been widely exploited as a means of selective delivery of therapeutic agents to the organelles. The ER signal we exploited is a 16-amino acid long; RQIKIWFQNRRMKWKK corresponding to the third helix of the DNA binding domain (homeodomain) of Antennapedia, often referred to as Penetratin. The NLS sequence used is a 10-amino acid long sequence which contains the nuclear localization sequence VQRKRQKLMP of the transcription factor NF-κB,^53^ it has previously been shown to be effective in directing cargo to the mammalian cell nucleus.^45^

**Fig. 2:**
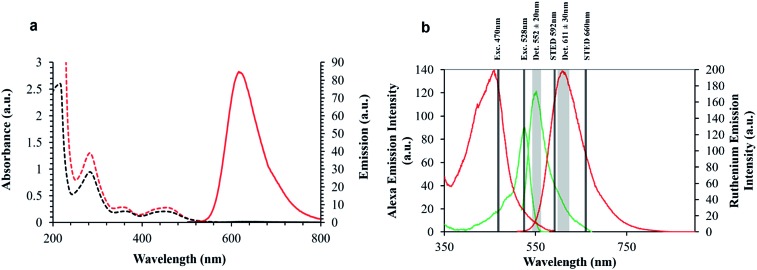
(a) Absorbance and emission properties of Ru-NLS in MeCN (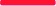) and water (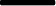) displaying the ‘light switch’ effect. (b) Spectral rationale of confocal and STED setup. Ruthenium excitation and emission (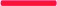), and AlexaFluor Fluor 532 excitation and emission spectra (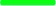), in aerated PBS solution.

The spectroscopy of the parent complex, **1** and arginine conjugate **2** are analogous to the ER peptide conjugate **3**, whose parent complex was reported recently,^48^ exhibit an electronic absorbance maximum at 454 nm which is assigned to the metal to ligand charge transfer (MLCT) transition, Fig. 2. Exciting into this absorbance feature, an intense emission band is observed centered at 602 nm with a quantum yield of 0.0316 ± 0.0015. Under aerated conditions, **3** exhibits a lifetime of 683 ± 8 ns in aqueous PBS (pH 7.4), which increases to 1.064 μs ± 14 ns (Table 1) after de-aeration with N_2_ for 15 minutes at room temperature. The optical absorbance of Ru-NLS is analogous, but because this complex contains the polyazaaromatic ligand^54^ dipyrido[3,2-*a*:2′,3′-*c*]phenazine it exhibits “molecular light switch” behaviour whereby as shown in Fig. 2, the emission from the complex, observed at 610 nm in acetonitrile, switches off in aqueous media, but it emits strongly when bound to DNA or membrane structures.^35,54–59^ The phenazine ligand was incorporated into this complex to promote DNA binding and associated selective emission from bound complex if the complex reaches the nucleus.

Also shown in Fig. 2, are the absorbance and emission spectra of AlexaFluor Fluor Phalloidin 532. AlexaFluor dyes are commercially available and recommended for STED because of their photostability.^22^

This probe was used here as a reference for the metal complex for STED as it is suited to depletion by the 660 nm STED laser and there is no cross talk such as energy transfer between the two dyes so they can be co-introduced into a single sample to gain insights into their relevant performance without mutual interference. The phalloidin bound AlexaFluor was used as it binds to actin filaments and is used here as a means of characterisation of the axial confinement of STED as a function of the luminophore identity. Overlap of the excitation and emission spectra of the probes and STED lines used are also indicated in Fig. 2.

### Cell uptake studies

**Fig. 3:**
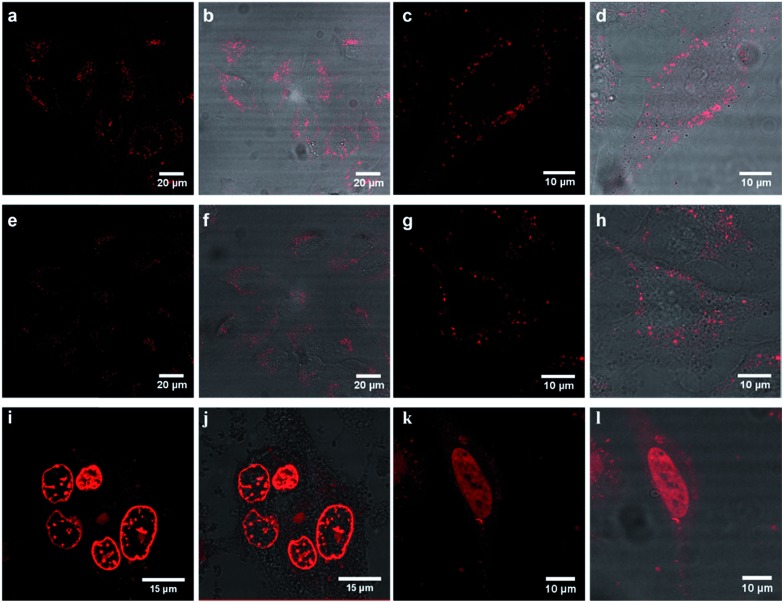
Confocal luminescence images of (a–d) Ru-ER, (e–h) Ru-Arg_8_ and (i–l) Ru-NLS in live HeLa cells where the ruthenium channel and overlay channel are shown for each complex. Cells were incubated in the absence of light with 70 μM Ru-ER and Ru-Arg_8_ for 4 h, and 40 μM with Ru-NLS for 24 h. The distribution of each complex is shown in a group of cells (column 1 and 2) and then focused on a single HeLa cell (column 3 and 4). Ru-ER and Ru-Arg_8_ were excited using a 488 nm white light laser and the emission was collected between 590 and 700 nm. Ru-NLS was excited using a 470 nm white light laser and the emission was collected between 565 and 700 nm.

The unconjugated parent complex, **1** [(Ru(bpy)_2_(phen-Ar)]^2+^ is impermeable to the plasma membrane.^48^ In contrast, as shown in Fig. 3, Ru-ER is taken up almost instantaneously into the cytoplasm of live HeLa cells. The cell uptake of Ru-ER was assessed across a range of conjugate concentrations between 10 and 100 μM in phenol-red free media by incubating Ru-ER in the absence of light with the HeLa cells at 37 °C with 5% CO_2_. 70 μM of the peptide conjugate was found to be the optimal concentration for uptake in terms of balancing imaging emission intensity with cytotoxicity, (see ESI Fig. S8†). By 2 h the complex had distributed throughout the cytoplasm excluding the nucleus, and by 4 h it had selectively localized within the endoplasmic reticulum (Fig. 3a–d). Uptake was similarly rapid for HeLa cells incubated with Ru-Arg_8_ (Fig. 3e–h) under the same conditions, with, based on image intensity, the same degree of uptake as the ER targeted probe.

However in contrast, the arginine conjugate distributed widely throughout the cytoplasm excluding the nucleus, with no specific organelle targeting over time. Ru-NLS uptake was similarly assessed in live HeLa cells. Remembering that this complex only emits when in a non-aqueous environment, *e.g.* bound to a membrane or intercalated into DNA, early stage uptake was more difficult to follow. Because of this it is difficult to follow uptake which thus appeared to be slower than for the ER or Arg_8_ however after less than 6 h incubation the emission could be seen from the membrane structures within the cell. After 24 hours the complex had both crossed the nuclear membrane and bound to the nuclear DNA within, as illustrated in Fig. 3i–l. To conclusively confirm signal peptide driven localization of Ru-ER to the ER and Ru-NLS to the nucleus, HeLa cells incubated with Ru-ER and Ru-NLS at 37 °C were co-stained with ER Tracker blue (1 μM final concentration) and DAPI nuclear stain (300 nM final concentration) respectively.

**Fig. 4:**
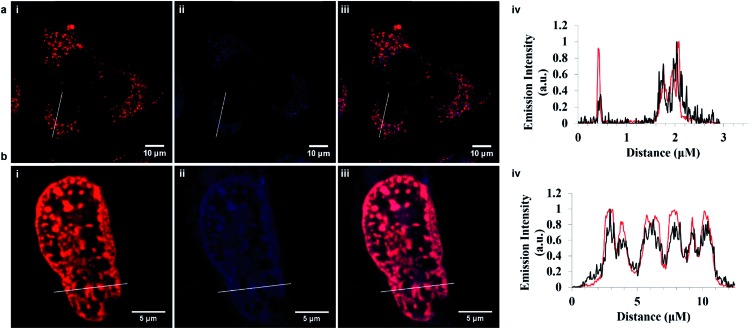
Cross-section confocal fluorescence image showing co-localization in live HeLa cells. (a) Ru-ER (70 μM) in red and ER Tracker Blue (1 μM) in blue shown and the corresponding plot demonstrating both compounds localize within the endoplasmic reticulum and are nuclear excluding, where (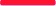) represents Ru-ER and (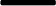) ER Tracker Blue. (b) Ru-NLS (40 μM) in red and DAPI nuclear stain (300 nM) in blue. The plot profile indicating both compounds co-localize within the nucleus, where (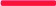) represents Ru-NLS and (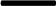) DAPI. Ru-ER was excited using a 488 nm white light laser and the emission was collected between 590 and 700 nm. Ru-NLS was excited using a 470 nm white light laser and the emission was collected between 565 and 700 nm. DAPI was excited at 633 nm and the emission was collected between 637 and 730 nm.

In both cases, Fig. 4, the peptide conjugates shown in the cross section plot in purple co-localized very precisely with the organelle stains shown in blue confirming the ER and NLS peptide directing sequences are both highly effective in directing the metal complexes to their respective organelles. The background luminescence from each complex outside of these regions is very low because of the selectivity of uptake, so excellent contrast is achieved. Indeed for Ru-NLS because of the dppz renders this a light switching probe, high contrast images of the chromosomes are evident from conventional live cell confocal imaging.

### STED imaging

**Fig. 5:**
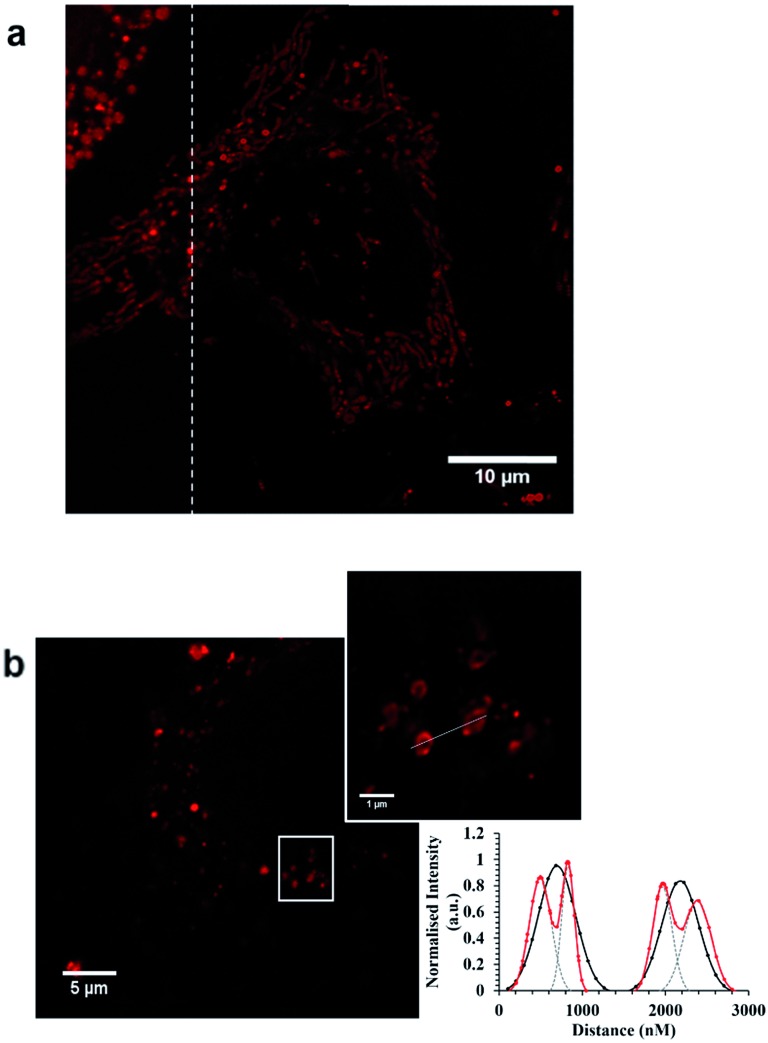
(a) STED image of Ru-Arg_8_ in a single HeLa cell. (b) Confocal and STED images of Ru-ER in a single HeLa cell demonstrating the tubular structure of the ER. The corresponding plot profile compares confocal (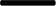) with STED (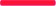). The fluorescence-intensity was fit to a Gaussian distribution (OriginPro) to obtain the separated distributions for the STED profile fitting, indicated by grey dashed lines. Both complexes were introduced to HeLa cells separately, for 4 h, at 70 μM in cell culture media. Samples were fixed with 3.8% paraformaldehyde for 15 minutes, and then mounted with Prolong Gold for 24 h before imaging. Both complexes were excited at 488 nm white light laser, and the emission collected between 590 and 700 nm. The 660 nm STED depletion laser was used for both complexes. Data is raw with no post-processing performed.

To evaluate the performance of the probes in STED imaging we prepared the HeLa cells following their incubation with the ruthenium peptide probes, by fixing them for 15 minutes using 3.8% paraformaldehyde and mounted using Prolong Gold. The samples were then imaged by both confocal fluorescence and STED microscopy using a Leica TSP DMi8 instrument. The depletion laser used for STED was a 660 nm line which has excellent overlap with the broad ruthenium ^3^MLCT emission centered at 611 nm. Fig. 5a shows representative STED images of a Ru-ER treated HeLa. The improvement in resolution is evident, wherein expanding the image in Fig. 5b, STED from the metal complex probes reveals the tubular structure of the smooth ER which is not resolved in the confocal images. This is also reflected in the resolution of the ER tubule structure shown in the *X*–*Y* plot shown in Fig. 5 which profiles two adjacent ER tubules. Under confocal imaging each tubule is resolved as a single spherical point fitted here to a Gaussian plot but in the STED image with the ruthenium peptide probe the hollow nature of the tubule is clearly resolved where the fluorescence intensity in the lateral direction reveals the walls of the tubules, the full width at half maximum (FWHM) of the imaged structures fitted here to a Gaussian distribution.

**Fig. 6:**
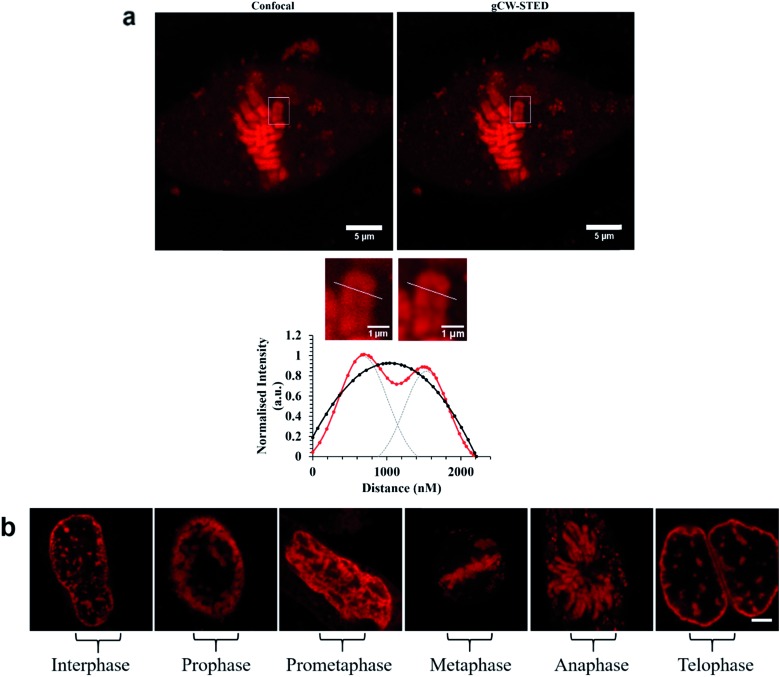
(a) Confocal and STED images of Ru-NLS bound to chromosomal DNA in the nucleus during metaphase. Bottom (a), line traces through a single chromosome (white) and the corresponding plot profile show the greatly improved resolving power of STED imaging (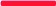) compared to confocal (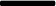). The FWHM was obtained by fitting fluorescence-intensity to Gaussian distributions (OriginPro). Two separated Gaussian distributions are indicated by grey dashed lines for the STED profile fitting. (b) STED images of Ru-NLS bound to DNA in the nucleus in fixed HeLa cells showing the different stages of cell division. HeLa cells were incubated with 40 μM complex for 24 h in the absence of light. The samples were fixed with 3.8% paraformaldehyde for 15 minutes, and then mounted with Prolong Gold for 24 h before imaging. Ru-NLS was excited at 470 nm white light laser and the emission was collected between 565 and 700 nm. The 660 nm STED depletion laser was used to acquire the STED images. Data is raw with no post-processing performed.

These images reveal both the capability of the metal complex as a STED probe enabled by the high precision labelling of the ER by the signal peptide modified probe. The targeting power of these peptide conjugated probes is further demonstrated in Fig. 6a which shows STED images of Ru-NLS in HeLa nuclei. As the complex behaves as a molecular switch on DNA binding, binding of the complex to chromosomal DNA is revealed with high contrast. Only the chromosomes and spherical structures which are slightly out of focus in these images are labelled. The latter are believed to be ribosomes bound to the nuclear envelope, their position at the outskirts of the nuclear envelope is confirmed by scanning the z-focus as shown in the video in ESI.†

**Fig. 7:**
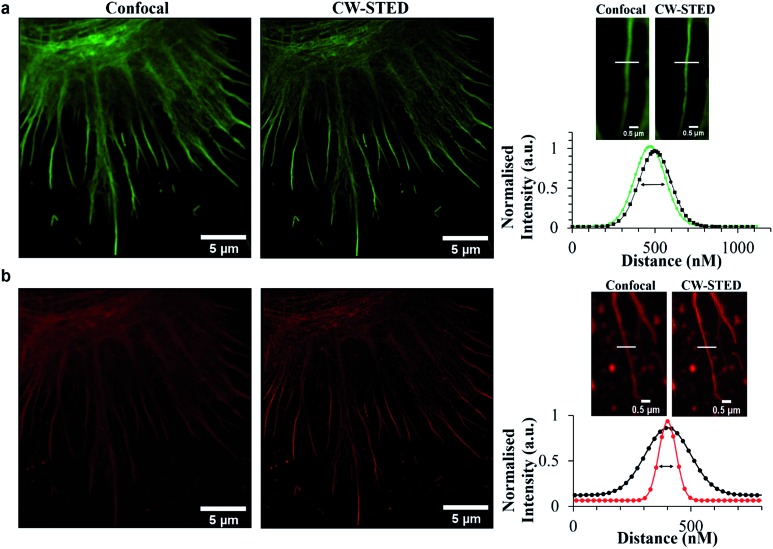
Comparison of confocal *versus* STED images of (a) AlexaFluor 532, and (b) Ru-Arg_8_. The FWHM was obtained by fitting fluorescence-intensity to Gaussian distributions (OriginPro). Images of fixed HeLa cells dual stained with Ru-ER and Alexa Fluor 532. Line traces and the corresponding plot profile show the greatly improved resolving power of STED imaging (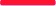) or (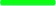) compared to confocal (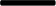). Samples were fixed with 3.8% paraformaldehyde for 15 minutes, and then mounted with Prolong Gold for 24 h before imaging. Samples were excited using a 488 nm white light laser, and the 660 nm STED depletion. Data is raw with no post-processing performed.

Emission from these latter spherical structures is attributed to binding of the DPPZ ligand to RNA mismatches within the ribosome structure.^60^ Single pair mismatches are a common structural motif in ribosomal RNA and DPPZ based Ru(ii) complexes have been shown in solution phase studies on RNA oligonucleotides to bind at single RNA mismatches by minor groove metalloinsertion with consequential emission switch-on at these sites.^61^ The remarkable contrast and resolution of the STED images permit with ease, assessment of the phase of cell division. In Fig. 5a the chromosome is shown aligning along the metaphase plate during metaphase in mitosis. Indeed the contrast possible with the molecular switch is excellent even in the confocal image. Nonetheless, the improvement in image contrast and resolution on STED imaging is striking with individual sister chromatids distinguishable as are grooves in the chromosome structures, possibly centromere sites. The relative improvement is reflected in the line profile taken through a single chromosome, where in STED each chromatid is resolved whereas in confocal such resolution is not achieved. As shown in Fig. 6b the light switch properties of Ru-NLS combined with STED allows facile identification of cell phase through a series of STED images selected from the nuclei of labelled HeLa cell during the different stages of mitosis. Interphase where the chromosomes replicate in the nucleus, alignment along the metaphase plate during metaphase, chromosome separation in anaphase and telophase where the chromosomes have split and become two new cells whilst the chromosome re-condense are all clearly distinguishable with outstanding clarity. Thus, peptide targeted ruthenium polypyridyl complexes are evidently well suited to STED, but to directly evaluate their performance against a conventional STED dye we compared Ru-ER with AlexaFluor Fluor Phalloidin 532. Whereas the 660 nm depletion line overlaps strongly with the ruthenium emission, this wavelength coincides with the red edge of the conventional AlexaFluor emission, as shown in Fig. 2. Red edge depletion is used in STED when using conventional organic probes to avoid re-exciting the fluorophore leading to decomposition or excitation into long lived dark state. We compared here the contrast and resolution achieved using each probe using both CW and time gated STED. Here, HeLa cells were dual-stained with AlexaFluor Phalloidin 532 and either Ru-ER or Ru-Arg_8_ and the cells were fixed following 4 hours incubation with the ruthenium complex. Cells were fixed for 15 minutes using 3.8% paraformaldehyde and mounted using Prolong Gold and were then imaged by both confocal and STED microscopy. Interestingly, dual staining with AlexaFluor 532, as shown Fig. 7 forces RuArg_8_ to enter the actin as well as the ER and other cellular organelles, which was useful as it allowed for evaluation of resolution over identical regions of the cell using a 2 colour experiment where each probe is excited independently; the ruthenium at 488 nm and AlexaFluor dye at 528 nm excitation and their emission detected at 552 and 611 nm with STED depletion carried out at 660 nm in each case. Fig. 7a compares the *x*–*y* profile of a confocal and STED image of AlexaFluor 532 at an actin filament, showing the full width half maxima of the data plot of the point spread function is used to evaluate and compare image quality between the confocal image and STED image. Fig. 7b compares the *x*–*y*-profile of the confocal and STED images of Ru-Arg_8_.

In the case of Ru-Arg_8_, the FWHM was improved by more than 60% from 226.66 nm in the confocal image to 86.8 nm with CW STED scan. In comparison for AlexaFluor STED the FWHM reduced by only 10%. The poorer performance of AlexaFluor compared with ruthenium under these conditions can be attributed to greater the overlap of the depletion line with the emission of the ruthenium. The 660 nm depletion is lies right at the red-edge of the AlexaFluor 532 emission but overlaps strongly with the ruthenium emission. Such strong overlap is rendered possible by the very large Stokes shift of ruthenium dye. To compare the performance of AlexaFluor when the overlap of its emission with the STED laser is closer to that used for ruthenium we also used a 590 nm STED laser to deplete this probe. However, as shown in ESI Fig. S11,† depletion using this line led to poorer resolution and poorer signal due to background noise consistent with the need for red-edge depletion with small Stokes shifted fluorophores in STED.

### Time gated STED

Time-gating the detection in STED microscopy can serve as a spatial filter, reducing background signal and the point-spread function of the interrogation volume to improve image resolution. In this method, a time delay is introduced after excitation to maximize the number of fluorophores within the depletion donut that are switched off. This approach increases resolution or decreases the STED intensity required for the same resolution, which is helpful in preserving probe integrity. The trade-off of time gating is that it reduces image intensity. However, imaging settings can be modified to increase the signal detected while at the same time increasing the resolution compared to continuous wave STED (CW-STED). Time gated STED (g-STED) is also a very useful approach to resolving 2 dyes with different excited state lifetimes and can also improve contrast by reducing contributions from background due to autofluorescence which is prevalent in biological samples.

**Fig. 8:**
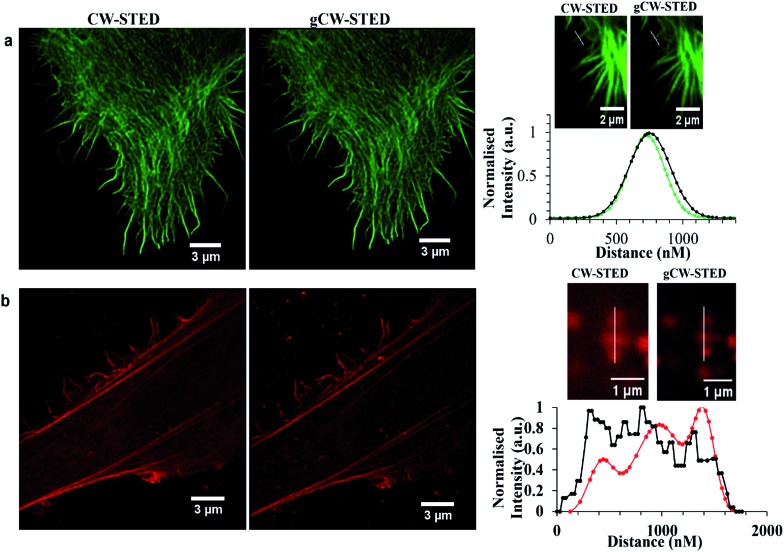
Comparison CW-STED *versus* gCW-STED images of Alexa Fluor 532 (a) and Ru-ER (b) in a fixed HeLa cell. Line traces and the corresponding plot profile show the greatly improved resolving power of using gating (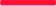) and (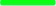) *versus* no gating (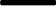). Samples were fixed with 3.8% paraformaldehyde for 15 minutes, and then mounted with Prolong Gold for 24 h before imaging. Samples were excited using a 488 nm white light laser, and the 660 nm STED depletion. Data is raw with no post-processing performed.

As described, Ru-ER has an excited state lifetime of 683 ± 8 ns in aerated aqueous media. Given the large discrepancy between the lifetime of this probe and the AlexaFluor we were interested in investigating the performance of the ruthenium probe in g-STED. And, in using CW-STED (g-STED) to evaluate if, when co-stained with the short lived AlexaFluor probe, contribution from this probe can temporally eliminated from the image, improving image quality by isolating signal only from the ruthenium probe only. Fig. 8 compares the CW-STED *versus* g-STED of AlexaFluor (a) and Ru-ER (b) in fixed HeLa cells. In both cases AlexaFluor 532 Tn was set to 0–3.5 ns while Tn was set to 3.5–12 ns for the ruthenium probe, where Tn is the lifetime window for data collection following the excitation pulse. As shown in Fig. 8b time-gating resulted in a much improved resolution of image Ru-ER in HeLa. This is well illustrated by the improvement in resolution of three adjacent features observed in the bottom right image in gSTED compared to CW STED, it is clear also that the background of images is dramatically improved in gSTED using a ruthenium complex as probe. Whereas, conversely the AlexaFluor FWHM dis-improved slightly increasing from 218.93 nm to 225.57 nm for same actin filament when in gCW-STED mode which is attributed to decrease in signal to noise ratio as some of the emission intensity is gated out for this short lived probe.

### Photostability and cytotoxicity

**Fig. 9:**
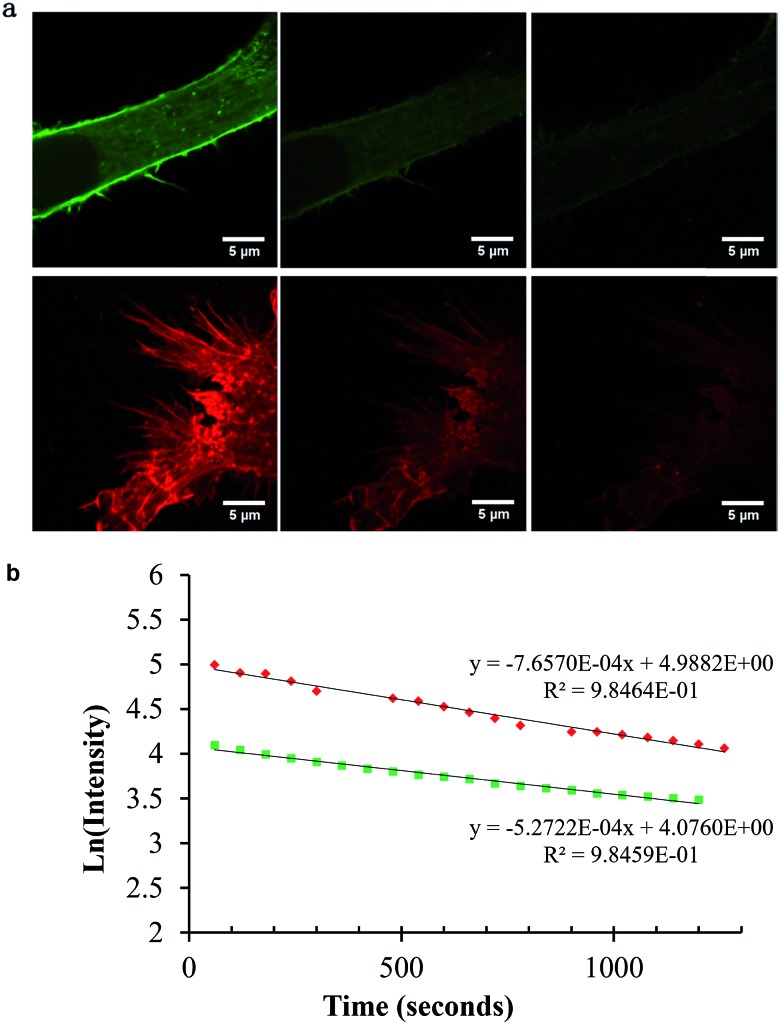
Photostability of AlexaFluor Phalloidin 532 (top) and Ru-ER (bottom) after 1, 15, and 30 frames of STED images acquired (a), and the corresponding emission intensity plot over the 30 frames (b). AlexaFluor Phalloidin 532 was excited at 528 nm white light laser, and the emission collected between 534 and 675 nm. Ru-ER was excited at 488 nm and emission collected between 590 and 700 nm. The 660 nm STED laser was used to acquire STED images of both samples at 0.05 W. Bottom, Ln (emission intensity) *versus* time plots for photobleaching of each probe under identical STED imaging conditions.

An important consideration in STED imaging is probe photostability. As described, the high intensity of the depletion laser makes demands on probe photo/thermal stability beyond those of conventional fluorescence imaging. The AlexaFluor dyes are widely recommended for STED imaging because they exhibit excellent photostability in comparison to many organic fluorophores. We compared the photostability of Ru-ER to that of AlexaFluor 532 in fixed HeLa cells by monitoring their bleaching under identical conditions. The cells were imaged under STED conditions continuously for 30 minutes with a pixel dwell time of 2.43 μs at 0.05 W incident laser power to assess for photo bleaching. Fig. 9a shows STED images collected after 60 seconds of continuous irradiation and then 15, and 30 minutes. Fig. 9b shows a corresponding log plot of the emission intensity values taken from a selected area at each frame for [(Ru(bpy)_2_-phen-Ar)-ER]^9+^ and AlexaFluor 532. As expected, the emission intensity of both probes diminishes over the course of the experiment under these rather harsh imaging conditions. Fitting the data to a simple first order plot we obtained rate constants for the photo bleaching process of 532 5.2 × 10^–4^ s^–1^ for AlexaFluor compared to 7.6 × 10^–4^ s^–1^ for Ru-ER indicating that under the same imaging conditions the photostability of the complex and AlexaFluor were comparable. However, it is important to highlight that in this experiment the STED depletion wavelength is far more closely overlapped with the ruthenium probe than the AlexaFluor 532 where it coincides only with the red tail of the latter. Thus given the weaker overlap of the excitation laser with AlexaFluor emission spectrum, the photostability of the ruthenium is at least as good as the AlexaFluor probe under STED imaging conditions.

Finally, the cytotoxicity of Ru-ER and Ru-NLS was considered in HeLa cells over a range of probe concentrations between 0.1 and 200 μm for 24 h in the dark at 37 °C using the Resazurin (Alamar blue) assay. As expected, neither parent complex; [(Ru(bpy)_2_-phen-Ar)-COOH]^2+^ nor [Ru(dppz)(bpy)(bpyArCOOH)]^2+^ induced toxicity, since neither enters the cells without peptide conjugation. Interestingly, it was found that Ru-ER was not toxic towards the HeLa cells with 97% of cells still viable following exposure to 100 μM of the complex, which is slightly more than our working concentration of 70 μM. While 72% of cells remained viable after 24 h exposure to high concentrations of 200 μM of complex. This contrasted with the Arg_8_ analogue where 0% of cells were viable after 24 h (ESI†). Fig. S8 in ESI† shows that Ru-Arg_8_ is highly toxic towards HeLa cells compared to Ru-ER where more than half remain viable after 24 h. Interestingly, the NLS conjugated complex also showed significantly less toxicity than the polyarginine complex with 55% of cells remaining viable following 24 hours incubation with 200 μM Ru-NLS complex. At our working concentration of 50 μM, 73% of cells remained viable following the 24 h incubation with the complex. This high viability allows for the nuclear DNA to be monitored and imaged over 24 h without inducing excessive damage towards the cells. We have previously shown that this complex conjugated to a mitochondrial targeting sequence was toxic towards HeLa cells at high concentrations, with 15% viable at 200 μM after 24 h.^48^ Although it has been noted previously that polyarginine can induce cytotoxicity we believe the variation in toxicity for the same complex with different peptides is likely to be a consequence of the precision of the localization of the complex with each peptide.^62^ Toxicity, is highest for the poly arginine which distributes the complex fairly ubiquitously, but for the nucleus, around the cell including into lysosome and mitochondria where in the latter in may be responsible for the observed cytotoxicity. Whereas, the ER and NLS peptide signals restricts the complex to the ER and nucleus respectively where it seems to exert little cytotoxic effect. Overall, within the working range of 50 to 100 μM a good balance is achieved between high quality images and good cell viability is achieved for both conventional confocal and STED imaging.

## Conclusions

We introduce ruthenium(ii) polypyridyl luminophores as a powerful new class of STED probe suited to both CW and time-gated STED modalities in cell samples. The ruthenium polypyridyl complexes have a range of photophysical characteristics that make them highly suited to STED. These include red, Stokes shifted emission, excellent photostability, low cytotoxicity and tunable environmental sensitivity. Their large Stokes shifts permit excellent matching of the stimulating depletion laser with their emission whilst avoiding anti-Stokes excitation. Their emission lifetimes make them particularly amenable to gated STED, giving a much wider window for gating than conventional organic probes. Using signal peptide conjugation, ruthenium(ii) complexes are targeted with very high precision to the endoplasmic reticulum and to the nucleus respectively to provide high resolution STED images of the tubular ER structure and of the dividing nucleus. Overall, the image quality and resolution achieved using Ru(ii) polypyridyl complexes as STED probes indicates that they are highly useful alternatives for super-resolution microscopy over current organic fluorophores. We hope this demonstration of their value in this domain will stimulate new approaches to design of probes for STED and related technologies exploiting metal luminophores. To demonstrate achievable resolution the samples used for STED here were fixed but there is no barrier, as demonstrated in the live cell confocal imaging presented, to applying these materials in live cell STED, so future work will likely explore their value in dynamic live cell studies. Furthermore, future work should also focus on improving the absorbance cross section and emission quantum yields of such complexes to improve their STED performance. Finally, as Ru(ii) complexes are largely red-orange emitters no doubt related metal complex luminophores with more tunable emission may prove useful also in this regard.

## Abbreviations

Arg_8_: Octa-arginine peptide sequence RRRRRRRR
Bpy: 2,2′-Bipyridine
CW-STED: Continuous wave stimulated emission depletion
DAPI: 4′,6-Diamidino-2-phenylindole nuclear stain
DNA: Deoxyribonucleic acid
Dppz: Dipyridophenazine
ER: Endoplasmic reticulum peptide sequence RQIKIWFQNRRMKWKK
FWHM: Full width half maximum
gCW-STED: Time-gated continuous wave stimulated emission depletion
HeLa: Human cervical cancer cell line
MLCT: Metal to ligand charge transfer
NLS: Nuclear localising sequence VQRKRQKLMP
PBS: Phosphate buffer saline
Phen: 1,10-Phenanthroline
STED: Stimulated emission depletion
R: Arginine
K: Lysine
I: Isoleucine
Q: Glutamine
L: Leucine
M: Methionine
P: Proline
W: Tryptophan
F: Phenylalanine
N: Asparginine
V: Valine
