# Supplementary material for: Precision targeted ruthenium(ii) luminophores; highly effective probes for cell imaging by stimulated emission depletion (STED) microscopy
Source: Chem Sci. 2016 Jun 30;7(10):6551–62. doi: 10.1039/c6sc02588a (PMC5131359; doi:10.1039/c6sc02588a)
Supplement: Supplementary file 1 [file SC-007-C6SC02588A-s001.pdf]

Electronic Supplementary Material (ESI) for Chemical Science.
This journal is © The Royal Society of Chemistry 2016

Peptide Targeted Ruthenium (II) Luminophores for Stimulated Emission Depletion (STED) Microscopy.

Aisling Byrne, Christopher S. Burke and Tia E. Keyes*.

School of Chemical Sciences and National Centre for Sensor Research, Dublin City University, Glasnevin, Dublin 9, Ireland.

*Corresponding author

Supporting data

Experimental

Synthesis - General Information.

Polypeptides (> 95 %) were purchased from Celtek Peptides (TN, USA) and used without further purification. All other reagents were procured from Sigma-Aldrich Chemical Co. The synthesis of dppz, bpyArCOOH, *cis*-Ru(DMSO)$_4$Cl$_2$, [Ru(bpy)$_2$-Phen-Ar-COOH]$^{2+}$, [Ru(bpy)$_2$-Phen-Ar-Arg$_8$]$^{10+}$ and [Ru(dppz)(bpy)(bpyArCOOH)]$^{2+}$ has been described previously.[1-5] ^{1}H NMR analysis was performed at 600 MHz using a Bruker Spectrometer and spectra were processed and analysed using Topspin software. The deuterated solvent was used for homonuclear lock and the spectra were calibrated against solvent peaks according to published values.[6] High Resolution Mass Spectrometry (HR-MS) analysis was performed at the HRMS facility, Trinity College Dublin.

General synthesis of the [Ru(bpy)$_2$-Phen-Ar-COOH] conjugates

A solution of [Ru(bpy)$_2$-Phen-Ar-COOH]$^{2+}$ (8 mg, 0.009 mmol) in DMF (1 ml) with (2-(1H-benzotriazol-1-yl)-1,1,3,3-tetramethyluronium hexafluorophosphate, HBTU (13.0 mg, 0.039 mmol) was stirred for 10 min at r.t. To this solution peptide (10 mg) in DMF (0.5 ml) with DIPEA (15 µL) were added and the mixture was stirred for 21 h. The solution was placed on ice and a saturated aqueous solution of potassium hexafluorophosphate was added to induce precipitation. After stirring for 10 min, the orange suspension was then filtered using 0.5 µm filter paper and the product washed with diethylether. The product was dissolved in minimum acetone and DCM was added dropwise to induce precipitation. The suspension was filtered and left under N$_2$ for 24 h to provide pure conjugate. An indication of reaction progress and purity was easily followed by TLC. (Silica. 90/10/1 MeCN/H$_2$O/KNO$_3$ (aq. satd.); R$_f$ (conjugate) = 0.00).

[Ru(bpy)$_2$-Phen-Ar-Arg$_8$]$^{10+}$ Ru-Arg$_8$

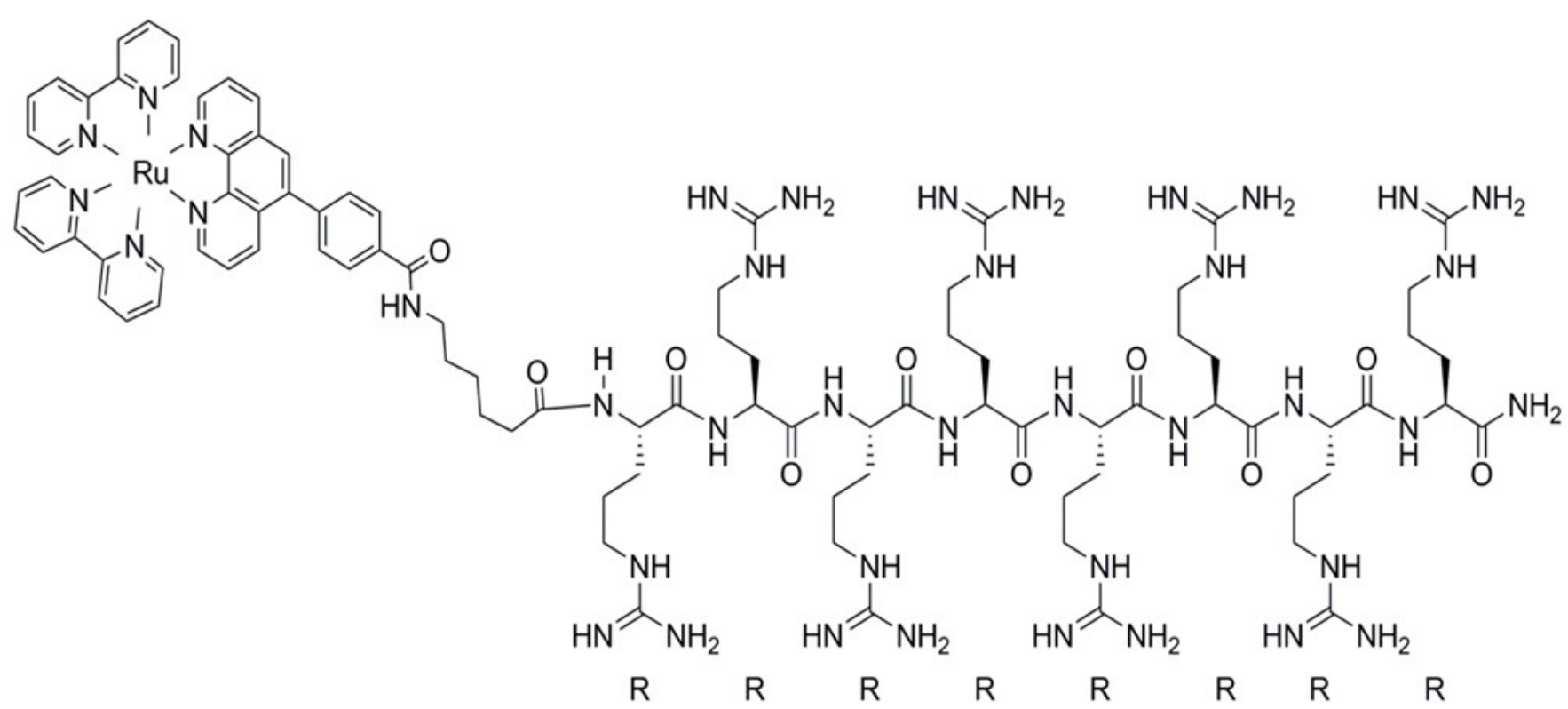

Characterisation of the octa-arginine conjugate was reported previously.[4] In the present work, additional HR-MS analysis confirmed formation of the Ru-peptide construct. HR-MS (MALDI (DCTB)-QTOF ES+): Found m/z ; 2220.0012 (Calcd. for $C_{93}H_{136}F_6N_{40}O_{10}PRu$ $[(M + PF_6)]^+$: 2220.0054).

$[Ru(bpy)_2$-Phen-Ar-ER$]^{9+}$ (Ru-ER)

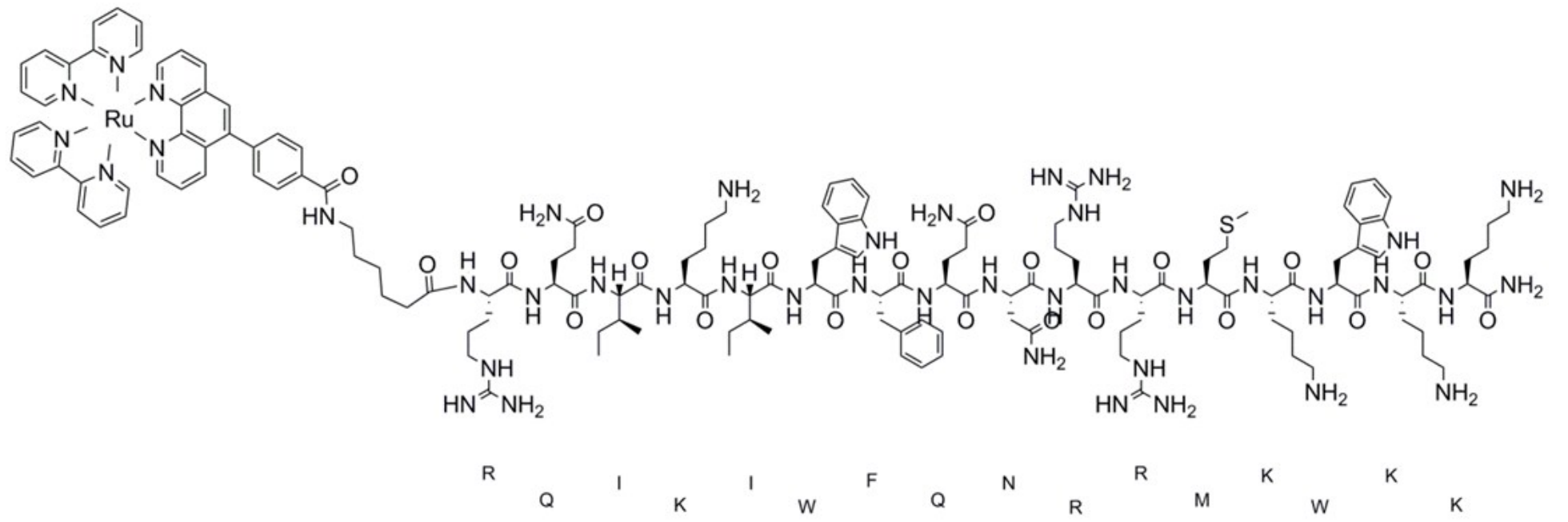

Yield: Red solid. ^{1}H NMR (600 MHz; Acetone-d_6) δ (ppm): 9.32 (m, 2 H); 8.94 (s, 1H); 8.82 (m, 1 H); 8.64 (m, 5 H, Ru-Ar-*H*); 8.30 (d, 1 H, Ru-Ar-*H*); 8.21 (m, 5 H, Ru-Ar-*H*); 8.12 (t, 3 H, Ru-Ar-*H*); 7.97 (s, 1 H, Ru-Ar-*H*); 7.88 (dd, 1 H, Ru-Ar-*H*); 7.80 (m, 2 H, Ru-Ar-*H*); 7.69 (dd, 2 H); 7.63 (d, 1 H, Ru-Ar-*H*); 7.57 (m, 3 H, Ru-Ar-*H*); 7.40 (m, 9 H); 7.18 (m, 3 H, Ru-Ar-*H*); 7.03 (m, 1 H); 6.88 (m, 2 H); 6.55 (m, br, 3 H); 6.36 (m, 1 H); 6.30 (q, 1 H); 6.21 (m, 10 H); 5.97 (m, 10 H); 5.56 (s, 1 H); 5.46 (t, 2 H); 3.6 – 4.5 (m, 16 H, peptide backbone H); 3.62 (s, 2 H); 3.47 (m, 6 H); 3.20 (m, 16 H); 2.68 (m, 8 H); 2.49 (m, 8 H); 2.28 (s, 4 H); 2.21 (t, 3 H); 2.14 (m, 5 H); 1.97 (s, 2 H); 1.94 (p, 2 H); 1.88 (m, 5 H); 1.71 (m, 14 H); 1.57 (s, 4 H); 1.3 – 1.5 (m, 21 H); 1.29 (s, 1 H). HR-MS (MALDI (DCTB)-QTOF ES$^+$): Found (Calcd.) *m/z* ; 1001.5954 (Calcd. for $C_{148}H_{203}N_{42}O_{21}Ru$ [(M - SMe (Met residue))]$^{3+}$: 1002.1717); 751.4481 (Calcd. for $C_{148}H_{203}N_{42}O_{21}Ru$ [(M - SMe (Met residue))]$^{4+}$: 751.6288).

Synthesis of the Ru-dppz NLS conjugate; [Ru(dppz)(bpy)(bpy-Ar-NFkB)]$^{6+}$. (RuNLS)

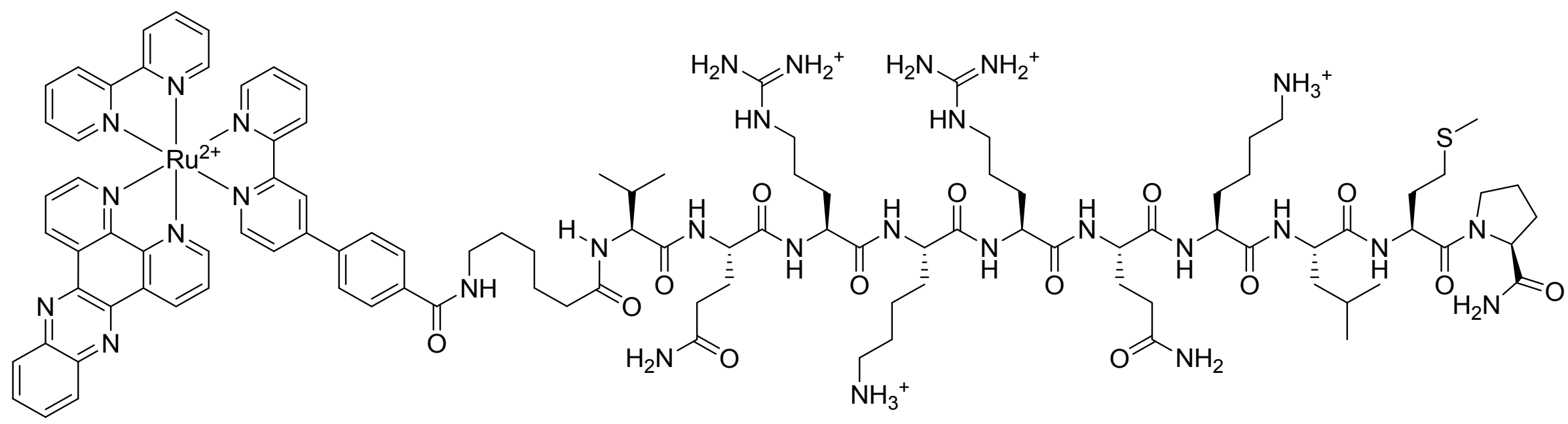

The NLS conjugate, [Ru(dppz)(bpy)(bpy-Ar-NFkB)]$^{6+}$, was synthesised by a HBTU coupling protocol analogous to that described above for the $[Ru(bpy)_2$-Phen-Ar-COOH$]^{2+}$ conjugates using in this case; [Ru(dppz)(bpy)(bpyArCOOH)]$^{2+}$ and the NLS sequence NH_2-Ahx-VQRKRQKLMP-$CONH_2$. Purification

was performed on preparative TLC plates (Silica-C18; 0.1 % TFA in 50/50 MeCN/H_2O as solvent). The product band was cleaved from the phase using methanol and concentrated under a nitrogen stream. The conjugate was precipitated by the addition of a few drops of sat. aq. ammonium hexafluorophosphate and was filtered and dried to afford the peptide conjugate as a red solid. ^1H NMR (600 MHz, D_2O drop in CH_3CN-d_3) δ (ppm): 9.66 (d, 2 H); 8.77 (m, 2 H); 8.57 (m, 2 H); 8.42 (m, 2 H); 8.08 – 8.25 (m, 5 H); 7.94 – 8.07 (m, 3 H); 7.90 (m, 6 H); 7.74 (m, 3 H); 7.50 (m, 2 H); 7.27 (m, 2 H); 5.34 (t, 1 H); 4.72 (s, 1 H); 4.26 (s, 2 H); 4.11 (s, 5 H); 3.87 (m, 1 H); 3.59 (s, 2 H); 3.31 (s, 2 H); 2.96 – 3.17 (m, 50 H); 2.87 (m, 14 H); 2.58 (m, 2 H); 2.30 (m, 5 H); 2.11 (m, 2 H); 1.97 – 2.07 (m, 6 H); 1.67 – 1.88 (m, 8 H); 1.57 (m, 12 H); 1.20 – 1.45 (m, 15 H); 1.17 (s, 1 H); 0.73 – 0.98 (m, 11 H). HR-MS (MALDI (CHCA)-QTOF) *m/z*: Calculated for $C_{106}H_{146}N_{30}O_{14}PF_6Ru$ [M + 4H (Basic Residues) + PF_6^-]$^+$: 2342.0035; Found: 2342.1399.

Characterisation Data for the Ru(II) conjugates

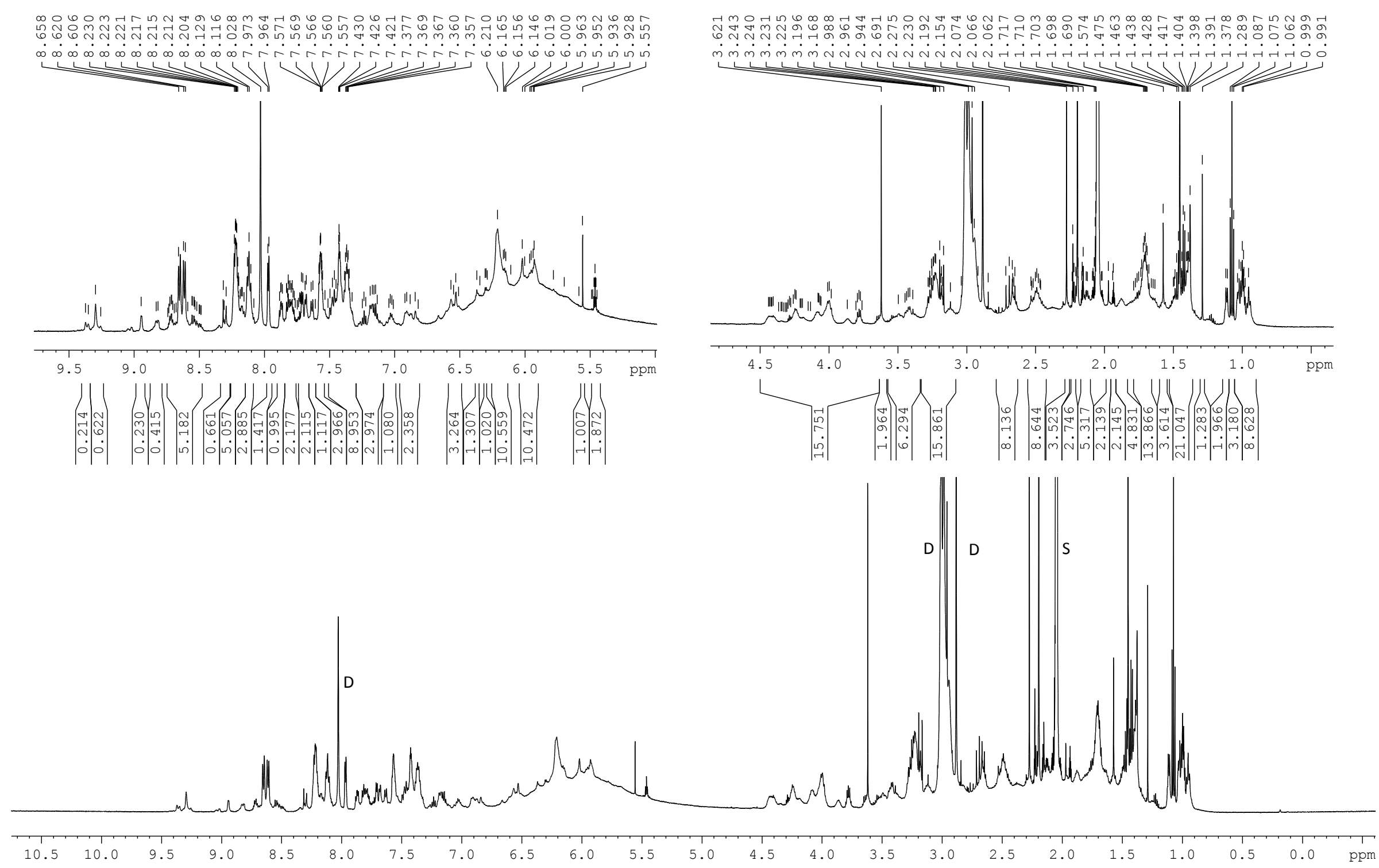

Figure S1: ^1H NMR (600 MHz) analysis of [Ru(bpy)$_2$-Phen-Ar-ER]$^{9+}$ in Acetone-d_6. S = Solvent peak; D = Residual DMF signals.

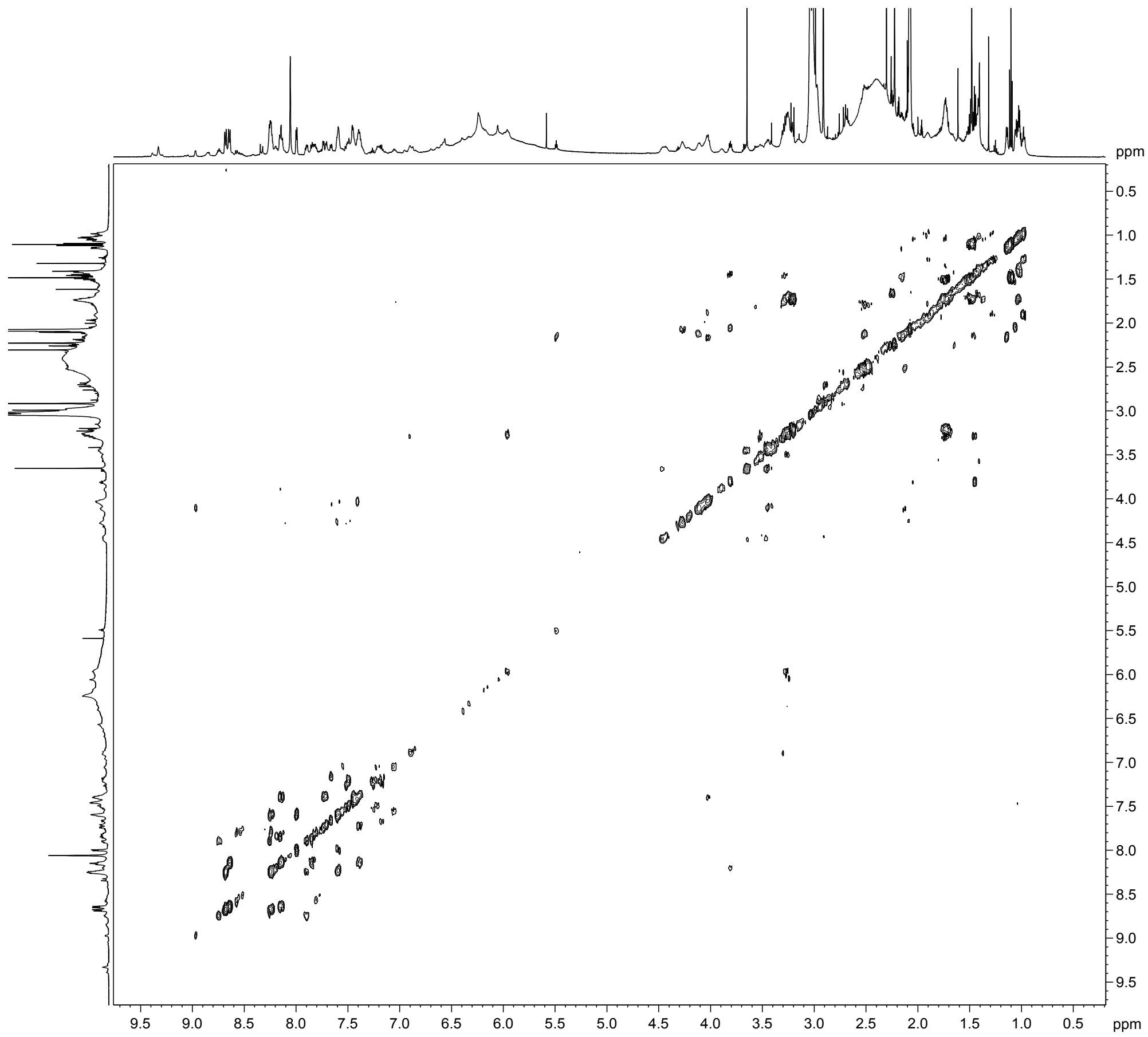

Figure S2: H-H COSY NMR (600 MHz) analysis of $[Ru(bpy)_2\text{-Phen-Ar-ER}]^{9+}$ in Acetone-d_6.

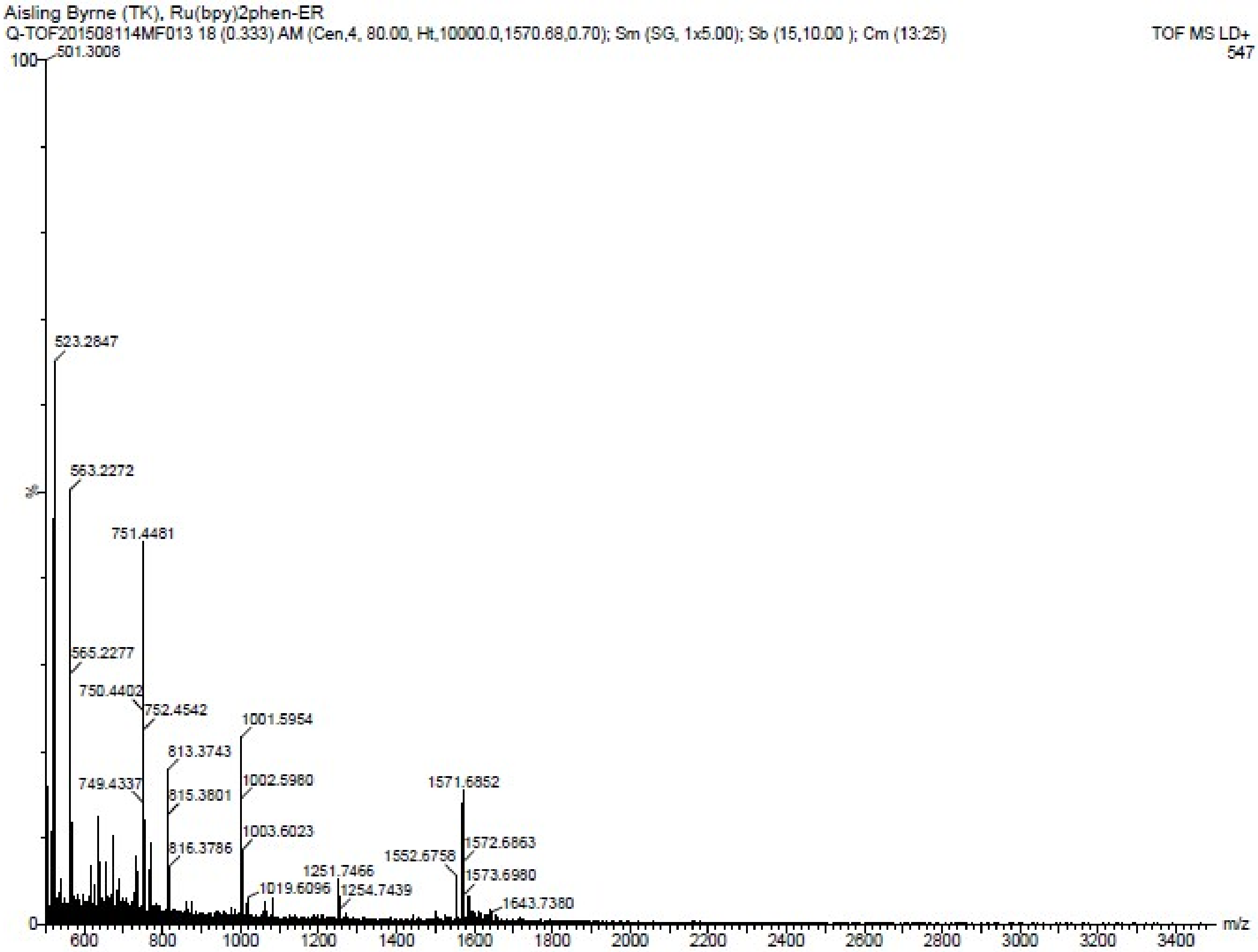

Figure S3: HR-MS analysis of $[Ru(bpy)_2\text{-Phen-Ar-ER}]^{9+}$ (RuER)

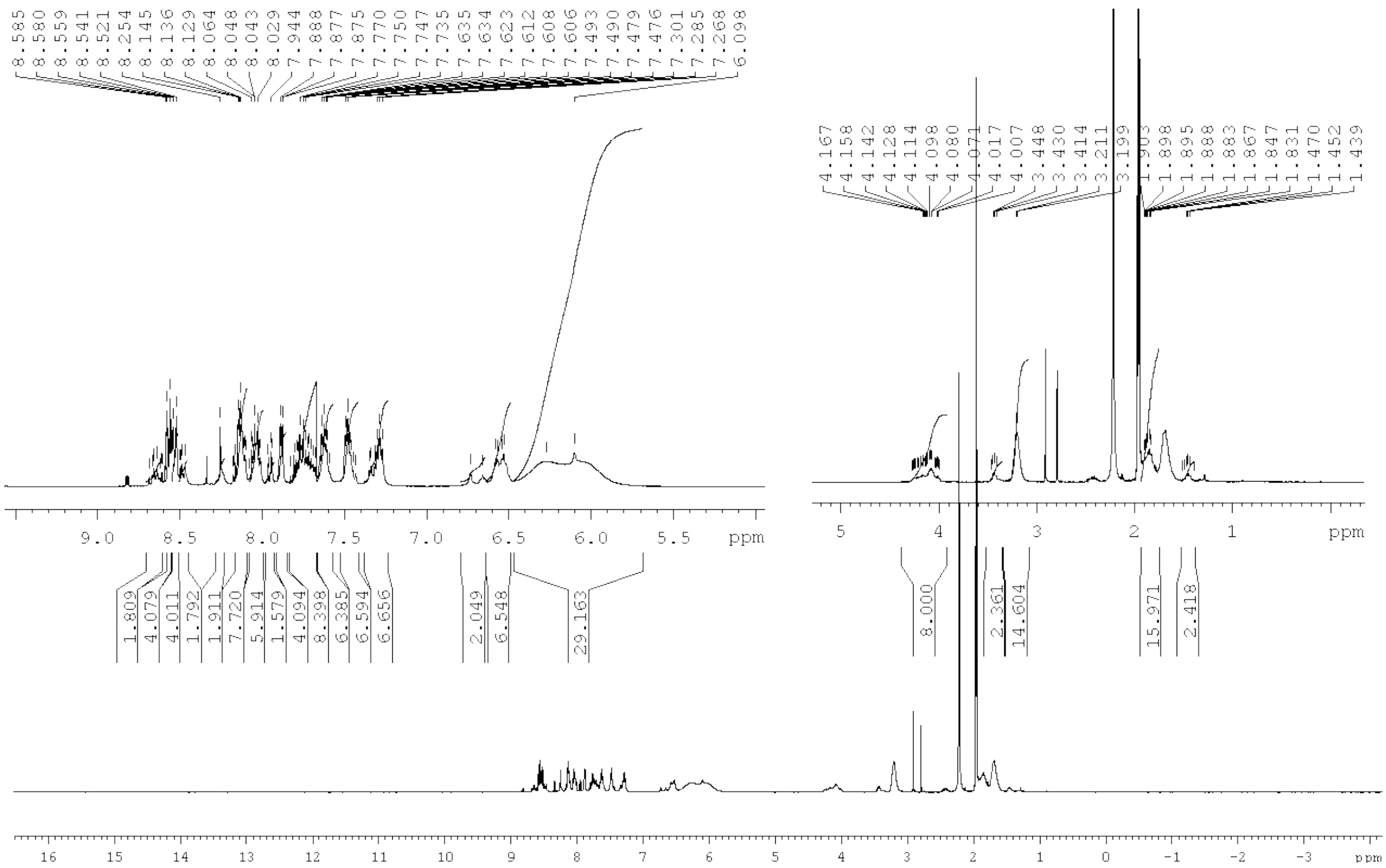

Figure S4: ^1H NMR (400 MHz) analysis of $[Ru(bpy)_2\text{-Phen-Ar-Arg}_8]^{10+}$ in CD_3CN.

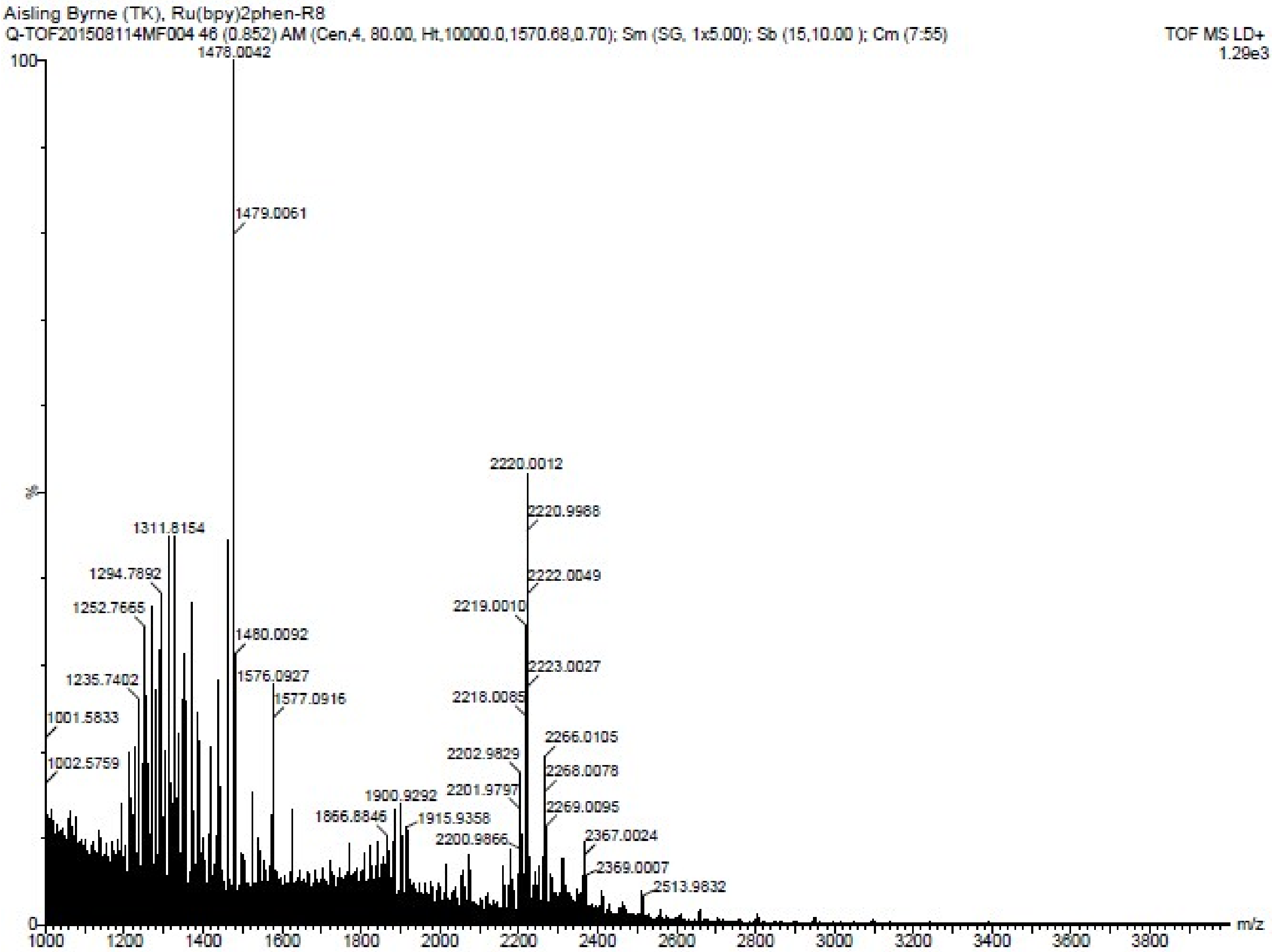

Figure S5: HR-MS analysis of $[Ru(bpy)_2\text{-Phen-Ar-Arg}_8]^{10+}$.

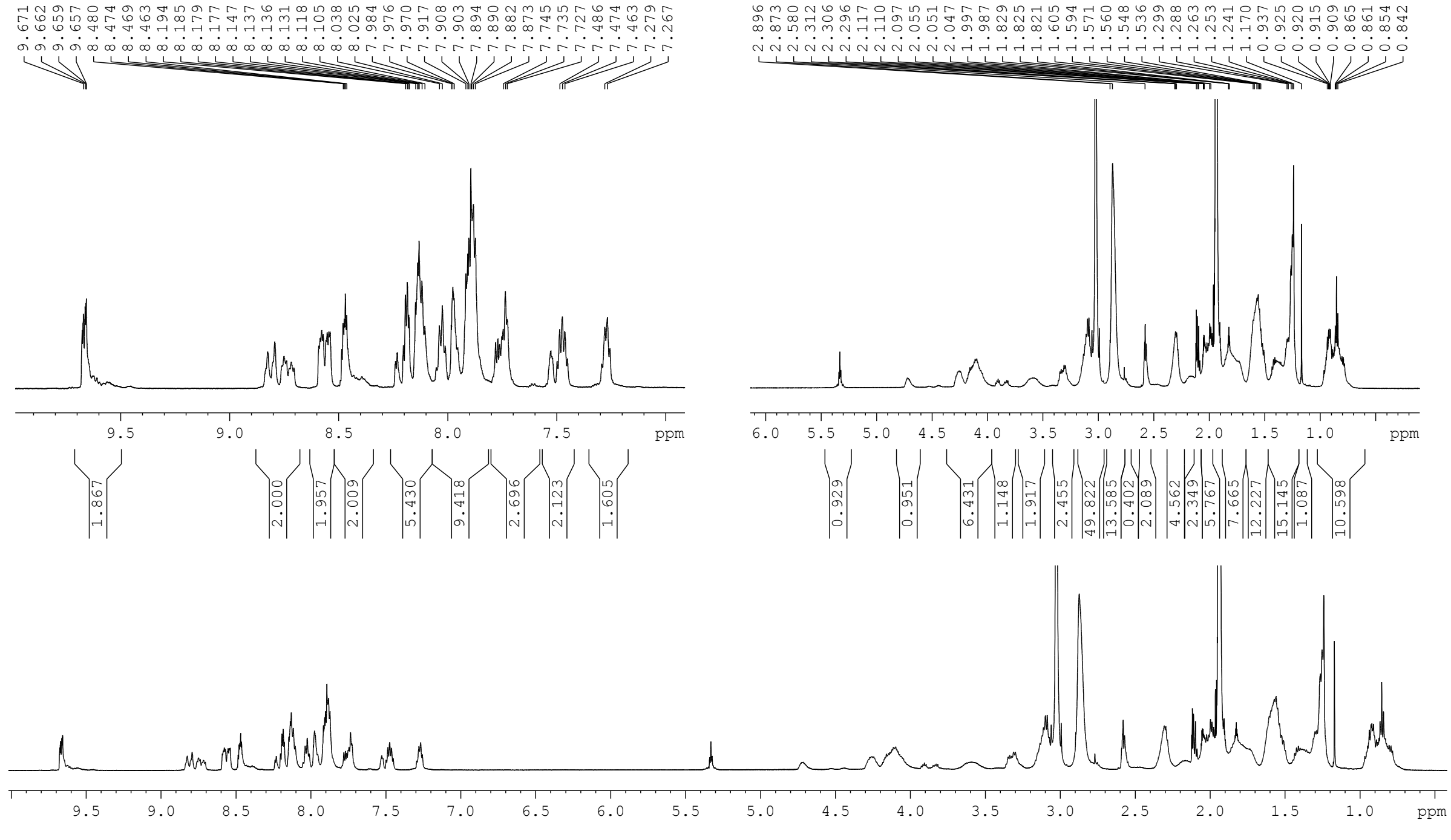

Figure S6: ^1H NMR (600 MHz) analysis of $[Ru(dppz)(bpy)(bpy\text{-Ar-NFkB})]^{6+}$ in CD_3CN/D_2O 99/1.

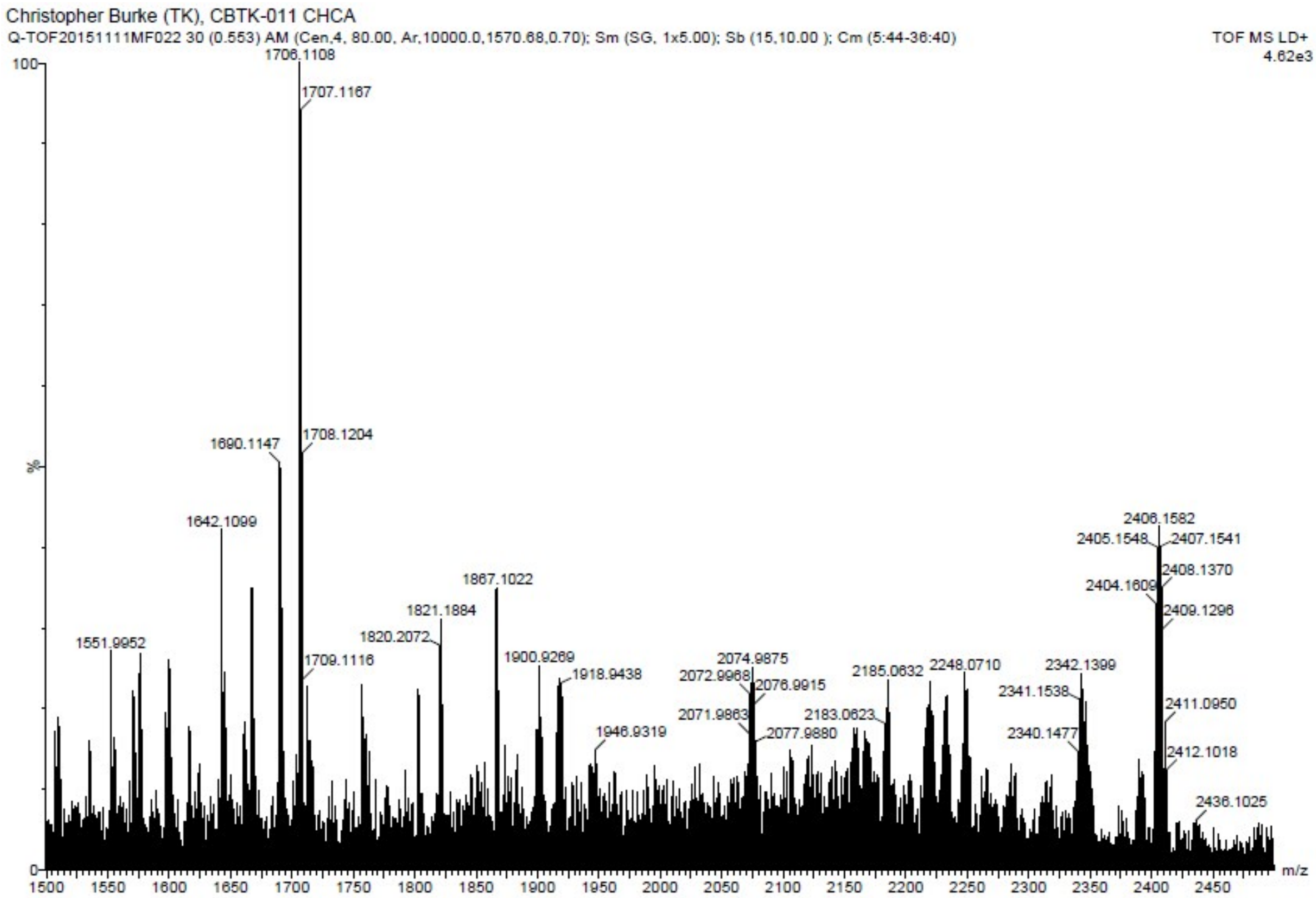

Figure S7: HR-MS analysis of [Ru(dppz)(bpy)(bpy-Ar-NFkB)]$^{6+}$ (RuNLS)

Table 1 – Summary of spectroscopic and photophysical data for parent and conjugated complexes.

Compound	Solvent	Abs<br><br>nm	Em<br><br>nm	Lifetime<br>Aerated<br>ns
$[Ru(bpy)_2\text{-phen-Ar-COOH}]^{2+}$	MeCN<br>PBS	452<br>456	601<br>608	150 ±6<br>455 ±11*
Ru-Arg8	PBS	453	601	579 ±11*
Ru-ER	PBS	454	602	683 ±8
$[Ru(dppz)(bpy)(bpyArCOOH)]^{2+}$	MeCN<br>PBS	282, 354, 454.<br>281, 358, 454.	620<br>None	228 ±1<br>-
Ru-NLS	MeCN<br>PBS	282, 354, 454.<br>283, 360, 451.	617<br>None	241 ±1<br>-

Notes: Solutions were prepared in MeCN using the PF_6^- salt of the complex whereas the Cl^- salt was preferred for the aqueous samples. All measurements were performed in triplicate using 10 µM solutions with the exception of the extinction coefficients where standard curves (10 – 30 µM) were used. PBS Buffer bought commercially at pH 7.4. Standard deviations are calculated from triplicate analyses. Lifetime (450 nm excitation, cut-off filter): Where applicable, bi-exponential decays were fitted with the fractional amplitudes provided in parentheses. All curve fitting conformed to chi squared tailfit criteria of $0.9 < \chi^2 < 1.1$. Slit widths set to 5 nm for emission and excitation runs. Deaeration was performed by bubbling N_2 through the analytical sample for 15 minutes.* values as marked were originally published elsewhere. [4,5]

Water Titration for Parent: $[Ru(dppz)(bpy)(bpy\text{-}Ar)]^{2+}$

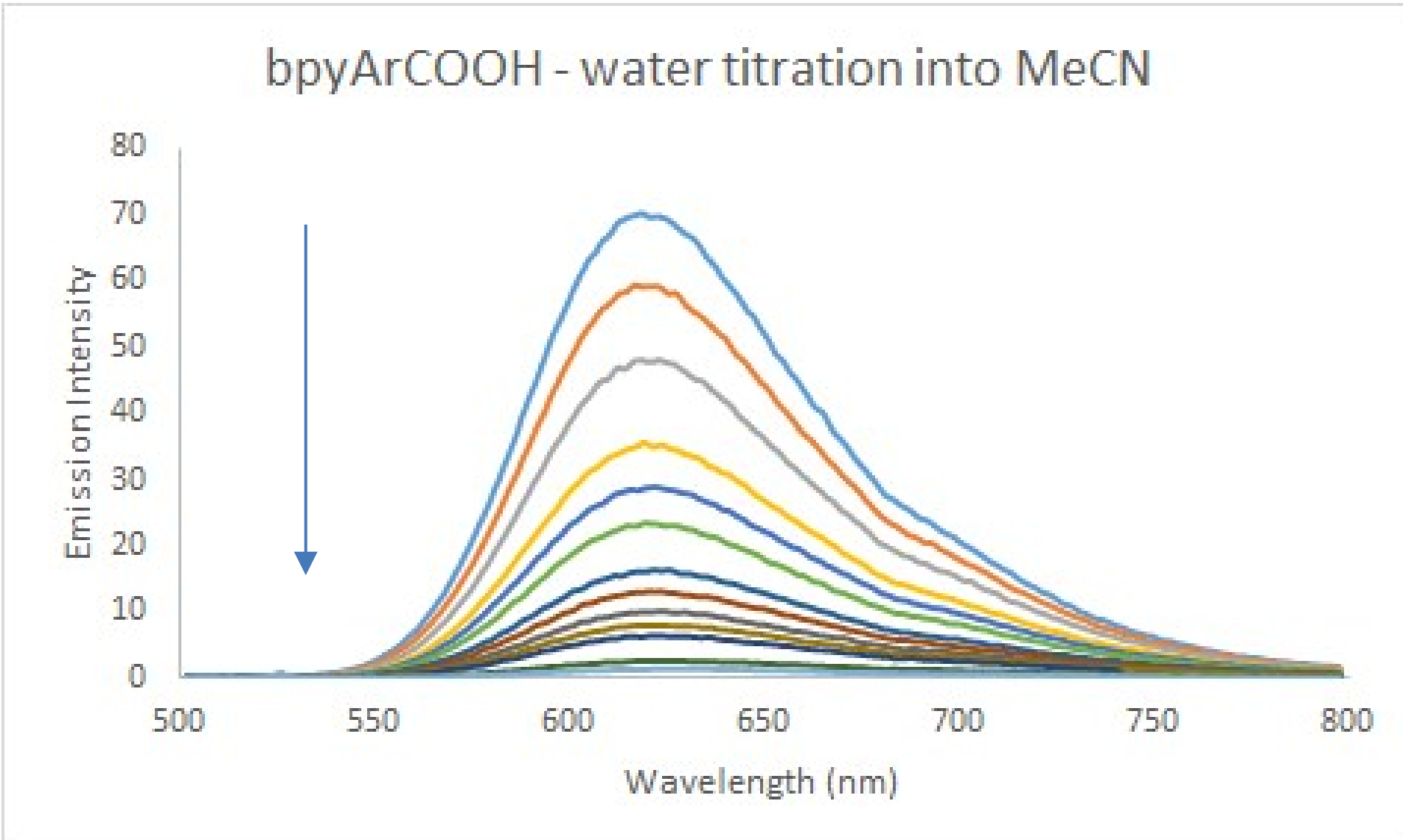

Figure S8 Demonstration of water switch effect for parent $[Ru(dppz)(bpy)(bpy\text{-}Ar)]^{2+}$ complex Emission spectrum of $Ru(dppz)(bpy)(bpy\text{-}Ar)]^{2+}$ in acetonitrile on addition of water: 3mL of a MeCN solution in a cuvette. To this, addition of aliquots of 30 uL (ca 1%) of water to show quench in presence of water. Approx 1 %V/V additions up to 10% water, then steps of 5 % up to 20 %. +4 % V/V water – half the emission quenched. 'Complete extinction of the $Ru(dppz)(bpy)(bpy\text{-}Ar)]^{2+}$ emission was observed at 20 % V/V water.

Real-time confocal luminescent imaging and STED microscopy Ru-ER

HeLa cells were seeded at 1.5 x 10^5 cells in 2 mL media on 35 mm glass-bottom culture dishes. Cells were grown for 48 h at 37ºC at 5% CO_2. The growth medium was removed and 70 µM of Ru-Arg_8 or Ru-ER in phenol red-free media was added and left to incubate for 4 h at 37ºC at 5% CO_2 in the dark. The dye/media solution was removed and cells were washed with PBS supplemented with 1.1 mM $MgCl_2$ and 0.9 mM $CaCl_2$. For live cell imaging, cells were imaged immediately using a Leica TSP DMi8 confocal microscope using a 100X oil immersion objective lens. A 488 nm white light laser was used to excite the complex, and emission was collected using a band pass 590-700 nm filter set. For fixed samples, HeLa cells were fixed using 3.8 % paraformaldehyde after washing with supplemented PBS for 15 minutes in the dark. The solution was removed and slides were washed twice with PBS. For Alexa 532 staining, 1:40 dilution of Alexa Fluor 532 with PBS was added to fixed HeLa cells for 30 minutes, removed and washed three times with PBS. Fixed cells were mounted using Prolong Gold.

To acquire STED images, a Leica DMI8 confocal system with STED lasers was used. A 488 nm white light laser was used to excite the ruthenium complexes collecting the emission between 590 -700 nm, and a 528 nm white light laser was used to excite Alexa 532 collecting the emission between 534 – 675 nm with a 100X oil immersion objective. Images were scanned at 1024 x 1024 resolutions using a scan speed of 0.01/s. A line accumulation of 6, and frame accumulation of 2 were used to eliminate as much background as possible. A time gating system was used to separate the two complexes. Alexa 532 was set to 0 - 3.5 ns, and ruthenium set to 5 –

12 ns. A 660 nm depletion laser was used to acquire the STED images, and images were accumulated twice for best signal. Images were deconvolved using Huygens Professional software. All data and FWHM analysis were carried out on raw images before deconvolution process using OriginPro.

Real-time confocal luminescent imaging and STED microscopy of Ru-ER

HeLa cells were seeded on a 35 mm glass bottom dish at 1.5 x 10^5 in 2 mL for 24 h at 37 °C at 5 % CO^2. Ru-NLS (40 µM) was added to the cells and incubated for 24 h in the absence of light. The complex was removed and the cells were washed twice with supplemented PBS. Cells were imaged using a Leica DMI8 confocal system. The complex was excited using a 470 nm white light laser and the emission was collected using a band pass filter 565 – 700 nm. Nuclear stain DAPI was added to the cells (300 nM) for 1 h prior to imaging. DAPI was excited using a 633 nm laser and the emission was collected btween 637 – 730 nm.

For STED imaging of Ru-NLS HeLa cells were seeded on poly-L-lysine coated 35 mm glass slides at 1.5 x 10^5 for 24 h. Ru-NLS was added for a further 24 h. the cells were washed with supplemented PBS. The cells were fixed with 3.8 % paraformaldehyde for 15 minutes and washed twice with PBS. The samples were mounted using Prolong Gold for 24 h before imaging. STED images were acquired by exciting with the 470 nm confocal laser line and a 660 nm STED depletion laser. Images were scanned at 512 x 512 resolutions. STED images were deconvolved using Huygens Professional deconvolution software.

Cytotoxicity assay

HeLa cells were seeded in a 96-well plate in 100 µL of media at 1 x 10^4 cells per well for 24 h at 37ºC with 5% CO_2. Either RuER, $[Ru(dppz)(bpy)(bpyArCOOH)]^{2+}$, Ru-NLS , or Ru-$Arg_8$ was added to give final concentrations of 200, 150, 100, 50, 20, 10, 1, 0.1 µM. Cells were incubated for 24 h at 37ºC at 5% CO_2. 10 µL of Resazurin (Alamar Blue) reagent was added to each well, and incubated for a further 7 h in the dark at 37ºC. The resazurin was converted to resorufin in viable cells and its absorbance was measured at 570 nm, with background measured at 600 nm using a Tecan 96-well plate reader.

Photostability

HeLa cells were seeded on poly-L-lysine coated 35 mm glass slides at 1.5 x 10^5 for 48 h. Ru-ER (70 µM) was added to the cells for 4 h at 37 °C and 5 % CO_2 in the absence of light. The cells were washed twice with supplemented PBS. The cells were fixed with 3.8 % paraformaldehyde for 15 minutes and washed with PBS. For Alexa 532 staining, 1:40 dilution of Alexa Fluor 532 with PBS was added to fixed HeLa cells for 30 minutes, removed and washed three times with PBS. Fixed cells were mounted using Prolong Gold. The samples were imaged using the optimum STED settings. A 488 nm white light laser was used to excite RuER , collecting the emission between 590 -700 nm, and a 528 nm white light laser was used to excite Alexa 532 collecting the emission between 534 – 675 nm with a 100X oil immersion objective. The STED 660 nm at 0.05 W was used for both samples. The images were acquired at 1024 x 1024 resolutions every 1 minutes for 30 minutes at a pixel dwell time of 2.43 µs. The emission intensity of a selected are in both samples was measured at each time interval over the 30 minutes and plot to show stability over time

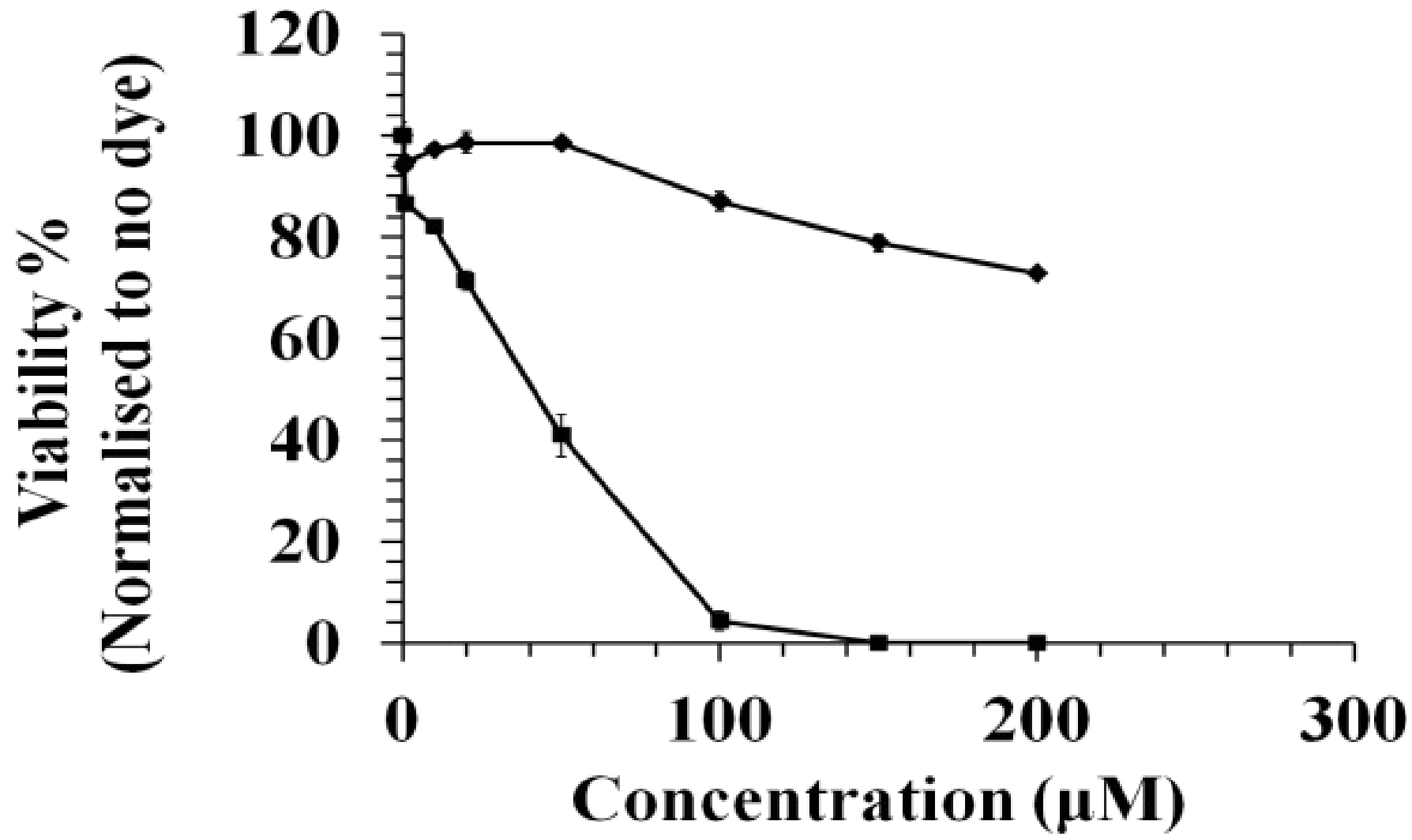

Figure S9: Cytotoxicity of Ru-ER (♦), and RuArg$_8$(■) at varying concentrations, incubated for 24 h in the dark at 37ºC at 5 % CO_2. HeLa viability was assessed using the Resazurin (Alamar Blue) assay (n=3).

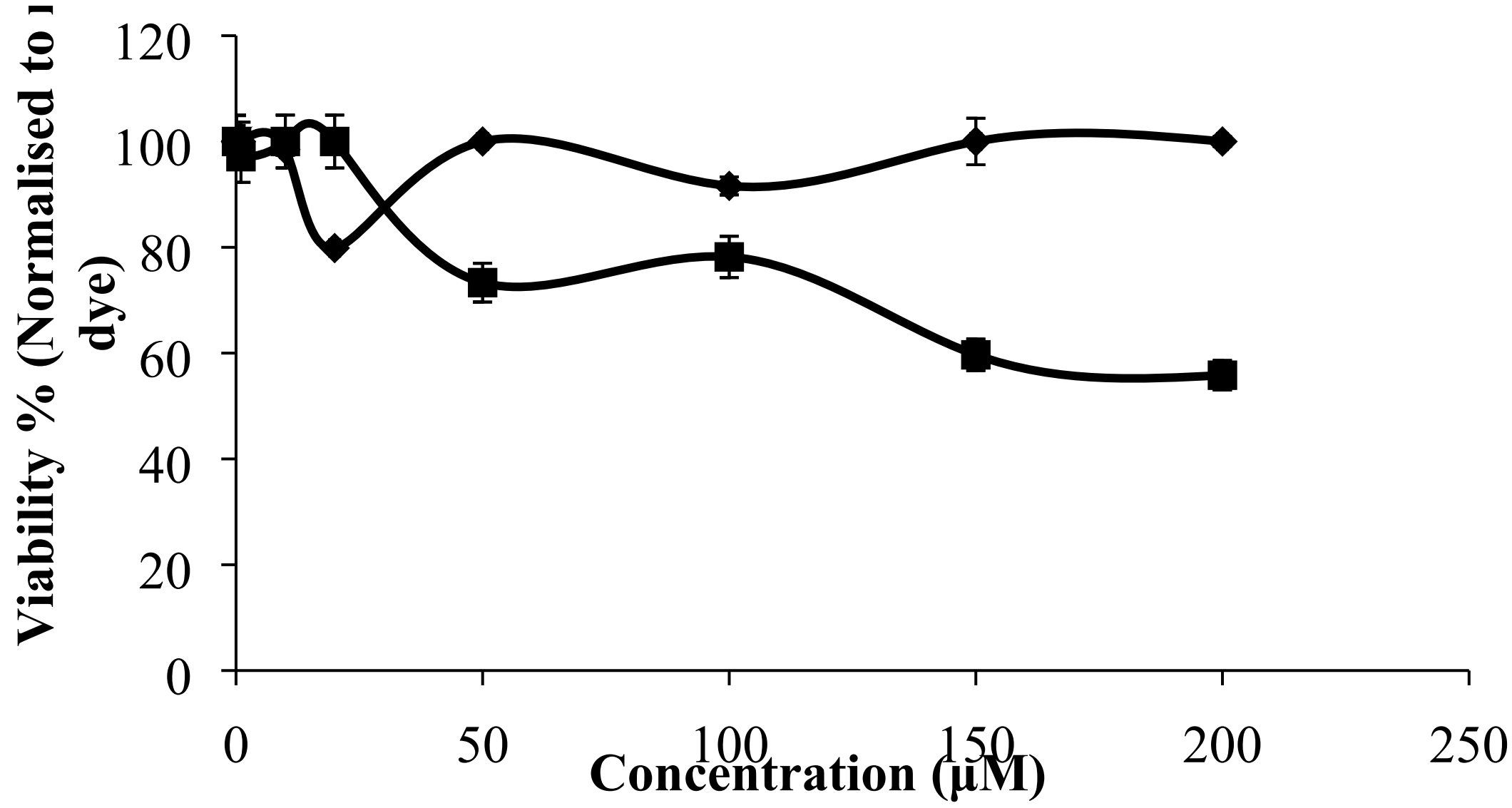

Figure S10 Cytotoxicity of parent: $[Ru(dppz)(bpy)(bpyArCOOH)]^{2+}$ parent complex (•), and RuNLS(■) in HeLa cells at various concentrations and left to incubate for 24 h at 37 °C in the absence of light. The resazurin (Alamar Blue) assay was used to determine the percentage (%) of viable cells remaining. (n = 2).

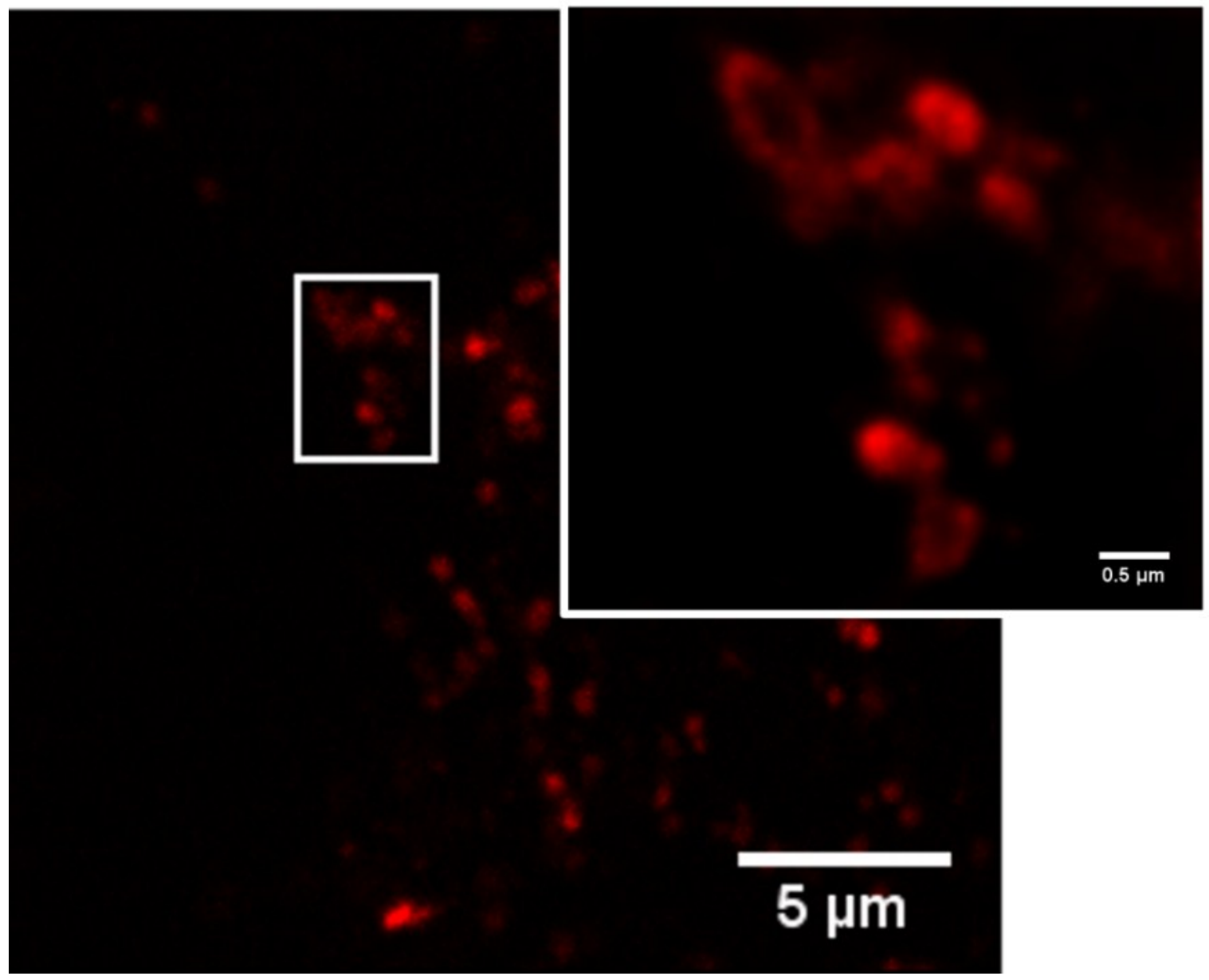

Figure S11 Confocal image of Ru-ER (70 µM for 4 h) in a fixed HeLa cell. Cells were fixed using 3.8 % parafamaldehyde for 15 minutes and mounted using Prolong Gold. The region of interest (ROI) highlighted demonstrates the STED image, giving more structural information on the ER. Ru-ER was excited using a 488 nm white light laser and the emission was collected between 590-700 nm. STED was carried out using the 660 nm depletion laser.

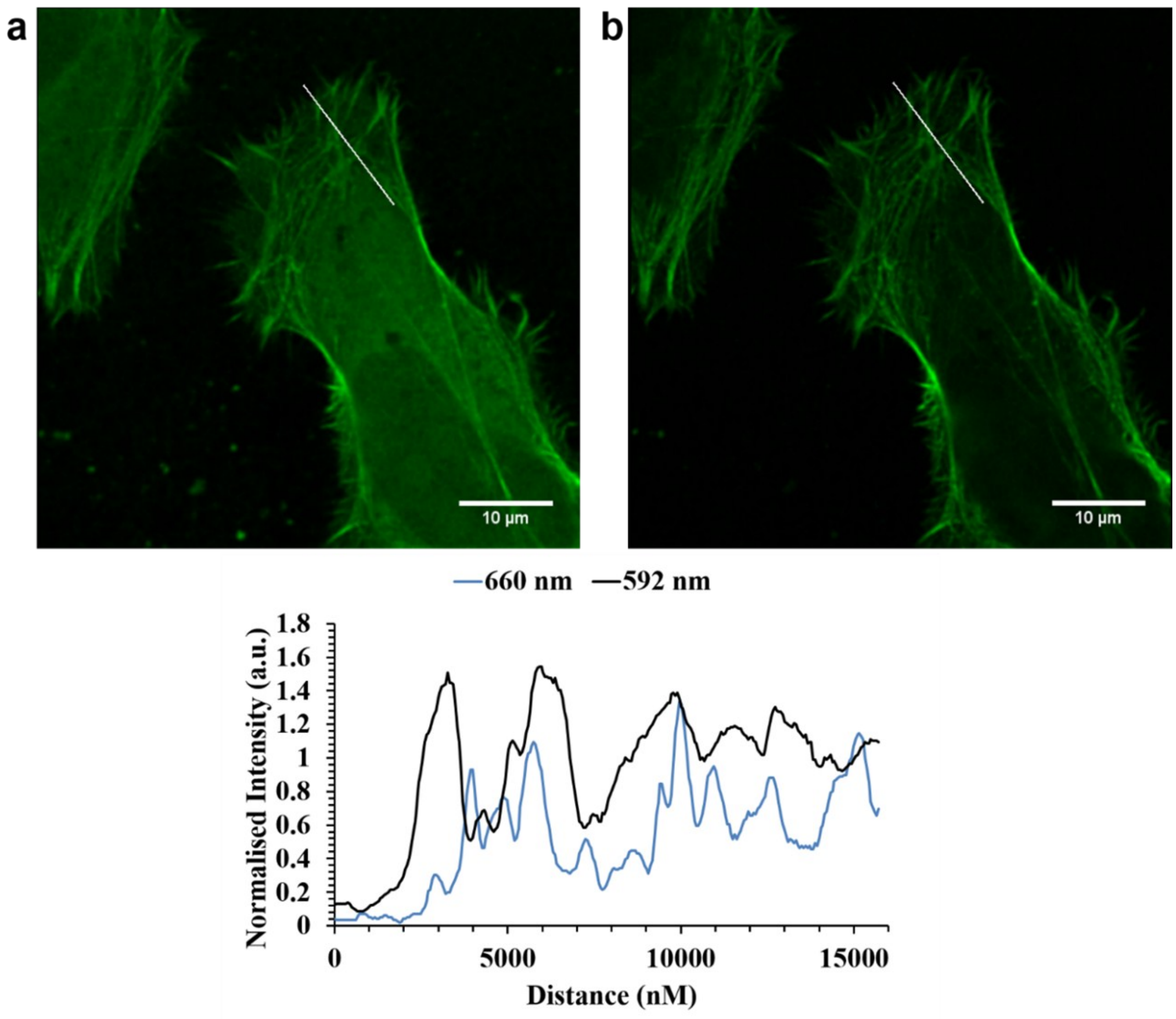

Figure S12 STED images of Alexa Fluor phalloidin 532 in fixed HeLa cell. Comparison of STED depletion laser (a) 592 nm and (b) 660 nm. Sample was fixed with 3.8 % paraformaldehyde and mounted using ProLong gold and excited using 532 nm white light laser. Line plot profile demonstrates that the STED 660 nm depletion laser gives optimum improvement compared to using the 592 nm laser.

References

1. Dickeson, J. & Summers, L. Derivatives of 1,10-Phenanthroline-5,6-quinone. *Aust. J. Chem.* **23,** 1023–1027 (1970).
2. Martin, A., Byrne, A., Dolan, C., Forster, R. J. & Keyes, T. E. Solvent switchable dual emission from a bichromophoric ruthenium–BODIPY complex. Chem. Commun. (2015).
3. Alston, J. R., Kobayashi, S., Younts, T. J. & Poler, J. C. Synthesis and characterization of rigid +2 and +3 heteroleptic dinuclear ruthenium(II) complexes. Polyhedron 29, 2696–2702 (2010).
4. Martin, A., Byrne, A., Burke, C. S., Forster, R. J. & Keyes, T. E. Peptide-Bridged Dinuclear Ru(II) Complex for Mitochondrial Targeted Monitoring of Dynamic Changes to Oxygen Concentration and ROS Generation in Live Mammalian Cells. J. Am. Chem. Soc. 136, 15300–15309 (2014).
5. Burke, C. S. & Keyes, T. E. An efficient route to asymmetrically diconjugated tris(heteroleptic) complexes of Ru(II). RSC Adv. 6, 40869–40877 (2016).

6. Fulmer, G. R. *et al*. NMR Chemical Shifts of Trace Impurities: Common Laboratory Solvents, Organics, and Gases in Deuterated Solvents Relevant to the Organometallic Chemist. Organometallics 29, 2176–2179 (2010).
